# Intracochlear distortion products are broadly generated by outer hair cells but their contributions to otoacoustic emissions are spatially restricted

**DOI:** 10.1038/s41598-021-93099-7

**Published:** 2021-07-01

**Authors:** Thomas Bowling, Haiqi Wen, Sebastiaan W. F. Meenderink, Wei Dong, Julien Meaud

**Affiliations:** 1 GWW School of Mechanical Engineering, Georgia Institute of Technology, Atlanta, GA USA; 2 VA Loma Linda Healthcare System, Loma Linda, CA 92357 USA; 3 Department of Otolaryngology - Head and Neck Surgery, Loma Linda University Health, Loma Linda, CA 92350 USA; 4 Petit Institute for Biosciences and Bioengineering, Georgia Institute of Technology, Atlanta, GA USA

**Keywords:** Cochlea, Hair cell, Inner ear, Computational biophysics

## Abstract

Detection of low-level sounds by the mammalian cochlea requires electromechanical feedback from outer hair cells (OHCs). This feedback arises due to the electromotile response of OHCs, which is driven by the modulation of their receptor potential caused by the stimulation of mechano-sensitive ion channels. Nonlinearity in these channels distorts impinging sounds, creating distortion-products that are detectable in the ear canal as distortion-product otoacoustic emissions (DPOAEs). Ongoing efforts aim to develop DPOAEs, which reflects the ear’s health, into diagnostic tools for sensory hearing loss. These efforts are hampered by limited knowledge on the cochlear extent contributing to DPOAEs. Here, we report on intracochlear distortion products (IDPs) in OHC electrical responses and intracochlear fluid pressures. Experiments and simulations with a physiologically motivated cochlear model show that widely generated electrical IDPs lead to mechanical vibrations in a frequency-dependent manner. The local cochlear impedance restricts the region from which IDPs contribute to DPOAEs at low to moderate intensity, which suggests that DPOAEs may be used clinically to provide location-specific information about cochlear damage.

## Introduction

The mammalian cochlea can detect sounds that cause vibrations of less than 1 nm, distinguish frequencies less than 0.4% apart, and operate over a wide dynamic range than spans a trillion-fold range of acoustic energy^1^. These amazing characteristics are commonly attributed to an active feedback mechanism linked to the nonlinear electromechanics of outer hair cells (OHCs)^2^. That is, sound-evoked vibrations in the cochlea stimulate mechano-electrical transduction channels located near the tips of OHC hair bundles (HBs), resulting in a modulation of the cell’s receptor potential. Due to these changes in OHC transmembrane potential, the abundantly present transmembrane protein *prestin* undergoes a conformational change, which exerts a force on the cochlear partition: a process called somatic electromotility. The prevailing hypothesis, supported by experimental data^3,4^, is that the somatic electromotile force delivers mechanical power to the traveling wave that propagates along the basilar membrane (BM), amplifying sound-evoked vibrations and improving frequency-selectivity and sensitivity in the healthy cochlea.

Because of the electromechanical feedback from OHCs, the cochlea generates sounds, called otoacoustic emissions (OAEs), that can be measured in the ear canal (EC). This article focuses on a specific type of OAEs, called distortion product OAEs (DPOAEs), which are measured at intermodulation frequencies when the ear is stimulated by two tones. DPOAEs are the summation of intracochlear distortion product (IDPs) generated due the nonlinearity of OHCs over a certain cochlear region. Once generated by OHCs, IDP traveling waves that result from the interaction of the cochlear partition with the intracochlear fluid in the scala tympani and scala vestibuli propagate within the cochlea. The IDPs in the scala vestibuli pressure at the base cause vibrations of the stapes and are transmitted to the EC by the middle ear, such that a distortion product is measured in the EC pressure as a DPOAE^5^. The IDPs have been measured and studied at different levels along the auditory pathway: perceptual (i.e.^6,7^), neural (i.e.^8^), and at the basilar membrane (BM) (i.e.^9,10^). DPOAEs are used clinically to screen for sensory hearing loss^11^, in addition to providing useful information about cochlear mechanics in research laboratories. However, where IDPs are generated within the cochlea and how these generated IDPs contribute to the ear-canal DPOAEs is not well understood.

While IDPs arise from the nonlinearity of OHC mechano-electrical transduction channels, in vivo recordings of IDPs in the electrical responses of OHCs are limited, i.e., forward propagation IDPs in cochlear microphonic were evidenced in scala media at the apical 2nd and 3rd turn of chinchilla cochleae (Gibian and Kim 1981). In addition, measurements of IDPs have been obtained from the mechanical response of the cochlea, i.e., in the BM vibrations^9,10,12^, intracochlear fluid pressure^13–15^ and more recently reticular lamina (RL) vibrations^16^. Characteristics of IDPs in the BM vibrations and fluid pressure, as well as theories of DPOAE generation^17–19^, suggest that only a relatively narrow region of the cochlea that is associated with the peak locations of the response to the primary tones contribute to the DPOAE. However, the recent measurements of the IDP response of the RL imply a potentially broad DPOAE generation region. This deviates from the current prevailing theories and would have important implications for the development of DPOAEs into an objective, noninvasive tool in the clinical diagnosis of location-specific hearing loss.

**Figure 1:**
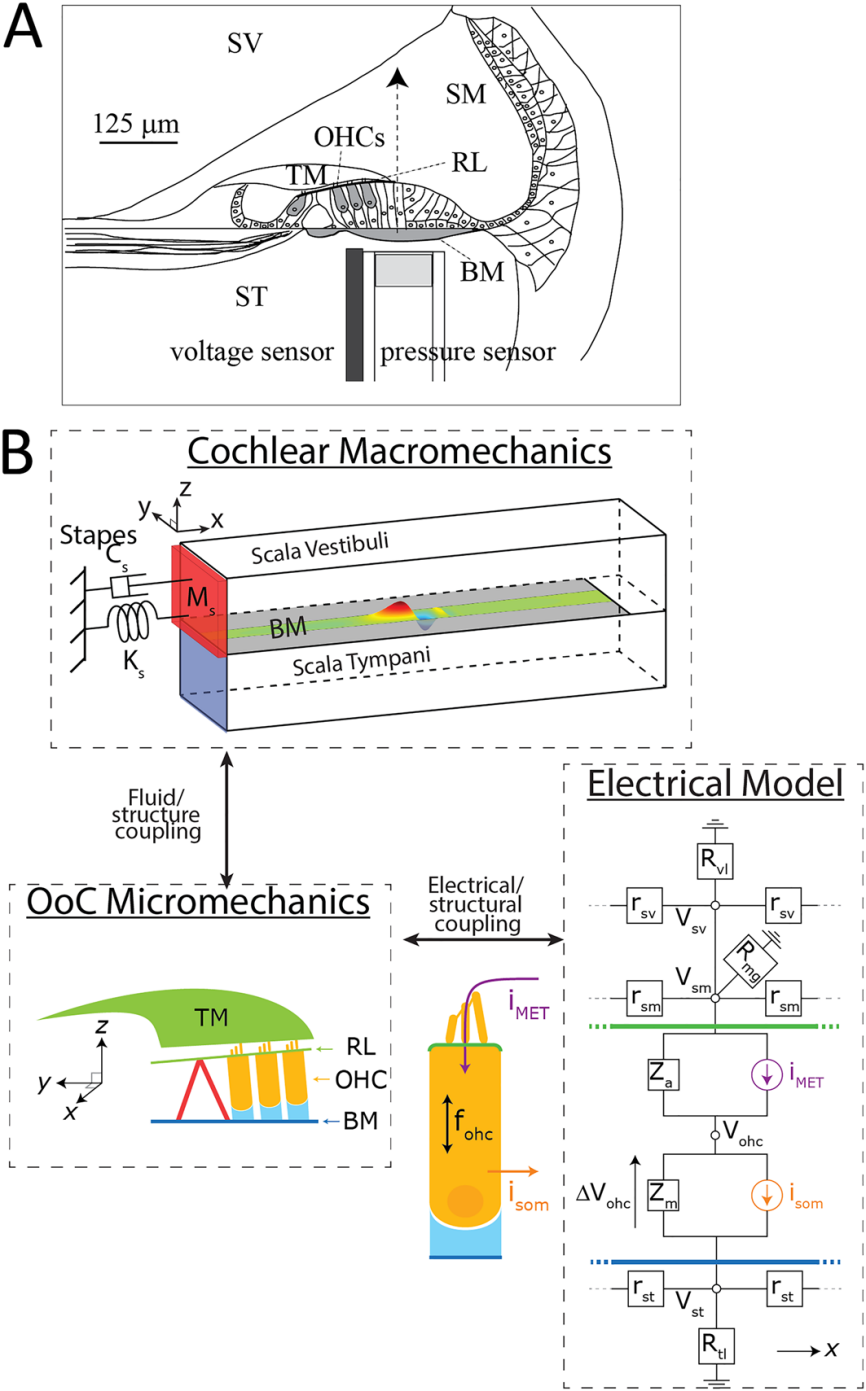
The electrical and mechanical responses of the cochlea to sound are studied using in vivo experiments and a computational model. (**A**) Schematics of the dual sensor probe used to measure fluctuations in the extracellular scala tympani (ST) potential, $V_{{st}}$ (referred to as the local cochlear microphonics, LCM, throughout the main text), and the acoustic pressure in the ST,${\text{~}}P_{{st}} ~$, at a location close to the basilar membrane (BM). (**B**) Schematics of the physiologically motivated computational model of the cochlea. The model includes a three-dimensional representation of fluid mechanics in the cochlear ducts with bidirectional coupling between the intracochlear fluid and the BM vibrations: the fluid in the ST and scala vestibuli (SV) applies mechanical loads on the BM; the BM vibrations are coupled to the ST and SV pressures via the linearized Euler equation. The BM is part of a micromechanical model of the organ of Corti (OoC) complex that also includes degrees of freedom for the tectorial membrane (TM), and active feedback from OHCs due to electromotility. This micromechanical model of the OoC is coupled to an electrical model that represents the electrical potentials in the SV, scala media (SM), OHC and ST. Vibrations of the OHC hair bundles due to the relative motions of the TM and reticular lamina (RL) result in a mechano-electrical transduction current, $I_{{MET}}$ (see Eq. (1)), which depolarizes the OHCs. In response to this change in the transmembrane potential,$~\Delta V_{{ohc}}$, OHCs generate an electromotile force, $f_{{ohc}}$ (see Eq. (2) in “Methods” section), which acts on the BM and RL. Due to the nonlinear relation between hair bundle deflection and transduction current, an IDP is generated when OHCs are stimulated by a two-tone stimulus. Longitudinal (x-direction) cables, of resistance per unit length $r_{{st}} ,~r_{{sv}}$ and $r_{{sm}}$ are included in the electrical model to represent the spreading of electrical current in the cochlear ducts; these cables are essential for the predictions of the key characteristics of the LCM in the experiments. Tables S1 and S2 in in Supplementary Information list the parameters seen in the Figure and provide the numerical values of these parameters.

This paper aims to determine the cochlear location(s) and extent of IDP generation by OHCs and to clarify how the regions of IDP generation contribute to the DPOAE measured in the EC. We report, for the first time, simultaneous measurements of IDPs in the electrical potential due to local OHC activity (electrical IDPs, eIDPs) and in the fluid pressure (fluid IDPs, fIDPs), both measured in the scala tympani (ST) near the BM using a dual sensor that consists of a microelectrode attached to a micro-pressure sensor^4^ (Fig. 1A). These in vivo intracochlear measurements are further interpreted by simulations using a computational model that provides a three-dimensional representation of the cochlear fluid mechanics with bidirectional mechanical and electrical coupling between the elements (Fig. 1B, also see “Methods” section, Supporting Material^20,21^). We show that eIDPs have a dual origin (distortion and linear components), which broadens the extent over which DPOAEs potentially originate. However, the ability of eIDPs, once generated, to give rise to fIDPs and to “escape” from their region of generation is limited by the mechanical properties (impedance) of the cochlear partition that supports OHCs. Only at relatively high stimulus intensities are fIDPs from a broad cochlear region able to propagate to the EC and contribute to the measured DPOAE; for low to moderate stimulus intensities our results indicate that the DPOAE is indeed generated within a narrow region associated with the frequencies of stimulus tones.

## Results

### Frequency dependent coupling between electrical and mechanical responses to single-tone stimulation

The generation of IDPs requires cochlear nonlinearity, which can be directly measured in the level-dependency of sound-induced mechanical and electrical responses of the cochlear partition.

**Figure 2:**
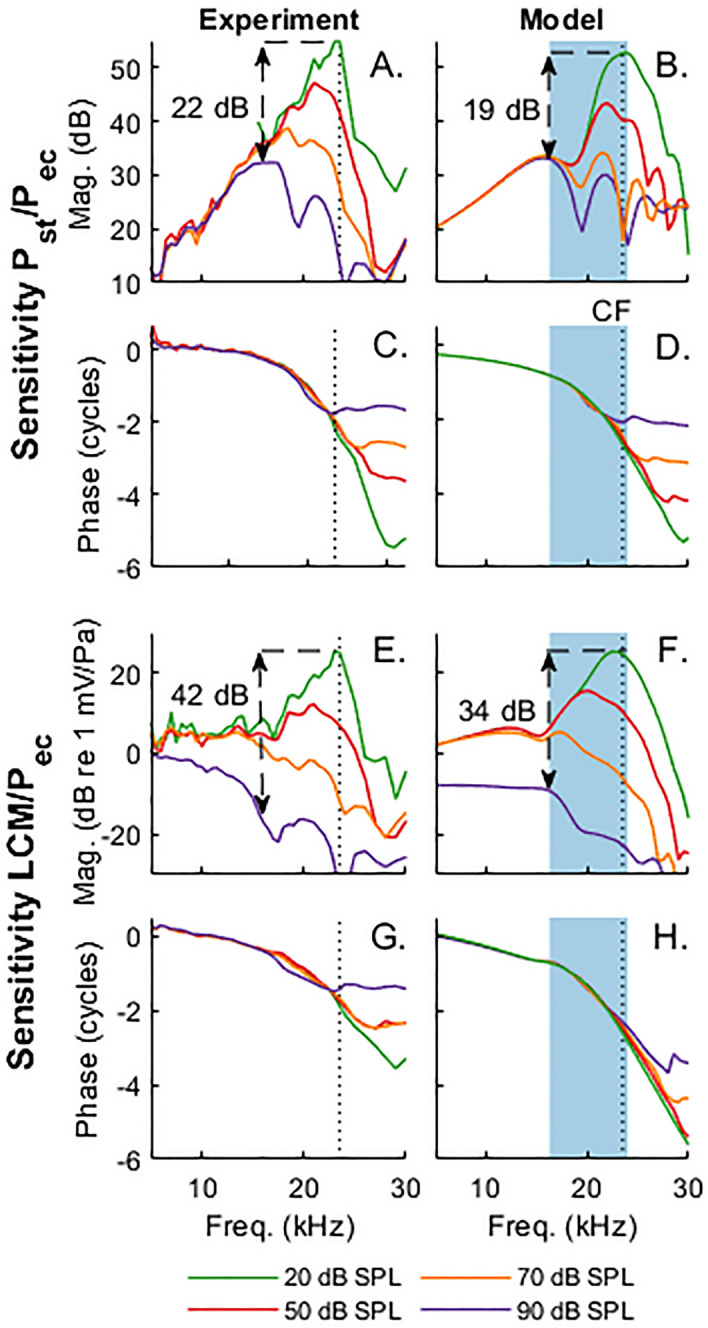
Comparison of animal experiments (left column, animal wg182) and model simulations (right column) for the mechanical (ST pressure) and electrical (local cochlear microphonics, LCM) responses of the cochlea to pure tone stimuli. In all panels, the vertical dashed line is the BF of 23.5 kHz. (**A–D**) Sensitivity (**A,B**) and phase (**C,D**) of ST pressure relative to EC pressure. (**E–H**) Sensitivity (**E,F**) and phase (**G,H**) of the LCM relative to EC pressure. The blue-shaded area in (**A,D,F,H**) corresponds to the frequency range where OHCs deliver significant power to the BM in response to 20 dB SPL tones. The vertical arrows identify the gain, defined as the ratio of the sensitivity at the BF at 20 dB SPL and the sensitivity at the frequency of maximum *P*_*st*_ sensitivity at 90 dB SPL.

The left column of Fig. 2 illustrates such level-dependency of the ST pressure, *P*_*st*_, and the electrical potential in ST measured at a location close to the BM in response to pure tones. The latter will be referred to as local cochlear microphonics, LCM, throughout the manuscript (e.g.^18^). As previously reported^4^, at low sound pressure levels (SPLs), *P*_*st*_ (Fig. 2A) and LCM (Fig. 2E) are tuned to the best frequency (BF) of the measurement location, 23.5 kHz at 20 dB SPL. Both increase with SPL in a compressive nonlinear manner such that their iso-intensity sensitivity curves (response magnitude normalized to EC pressure) fan out at frequencies around the BF, similar to what has been observed in the sound-evoked vibrations of the BM^22^. The phase responses of both *P*_*st*_ (Fig. 2C) and LCM (Fig. 2G) are consistent with a traveling wave that slows down as it approaches the BF. Differences between *P*_*st*_ and LCM are more pronounced at high SPL, where the LCM exhibits a low-pass response while *P*_*st*_ is broadly tuned, and the nonlinearity of the LCM extends to lower frequencies. The gain (defined here as the sensitivity difference between low SPL at the BF and at the frequency of maximum *P*_*st*_ sensitivity at 90 dB SPL) of the LCM is about 20 dB larger than that of *P*_*st*_.

The model (Fig. 1B) captures the key features seen in the experiments. While *P*_*st*_ is tuned at all SPLs (Fig. 2B), the LCM transitions from a tuned response at low SPL to a low-pass response at high SPL (Fig. 2F). Nonlinearity extends to the lowest frequencies at high SPL for the LCM, while it is limited to the BF region for *P*_*st*_. The gain of LCM is greater than that of *P*_*st*_, and both measures show traveling wave phase accumulation (Fig. 2D,H).

In our model, the nonlinear electromotile OHC force potentially acts on the BM over a broad extent. However, we found that the OHC force has limited effects on the BM vibrations, and thus *P*_*st*_, at low frequencies, the sub-BF region, because the force is negligible at these frequencies given the large BM impedance and mechanical load applied by the fluid pressure on the BM (Fig. S2). It therefore only delivers significant mechanical power to the BM in the frequency range highlighted by the shaded area in the simulations of Fig. 2 (see Supplementary Information for the calculation of the power). It is this limited range, of power amplification, from about 0.5 octave below to the BF, that is responsible for the sensitive and sharply tuned *P*_*st*_ responses around the BF at low SPL. The compressive nonlinearity observed in both *P*_*st*_ and LCM as SPL increases is due to the saturation of the mechano-electrical transduction channels and of the active feedback mechanism related to cochlear amplification.

In our model predictions, the LCM is a summation of OHC potentials that are generated over a broad spatial extent (Fig. S1 in Supplementary Information) due to the presence of a longitudinal electrical cable in ST (see Fig. 1B). However, contributions to the LCM from OHCs in the BF region tend to cancel out at the observation location because the wavelength of the traveling wave is small compared to the space constant of the ST electrical model. At all SPLs, this cancellation tends to broaden the tuning and decrease the magnitude of the LCM response around the BF (see Fig. S1B); this broadening makes the LCM low-pass only at high levels, when the tuning of the transduction current is very broad. At these high levels, the LCM responses would be broadly tuned if the ST cable were neglected (Fig. S1), which does not match the experiments, showing the importance of longitudinal electrical coupling to describe the low-pass response that is experimentally observed.

### Characteristics of eIDP and fIDP

**Figure 3:**
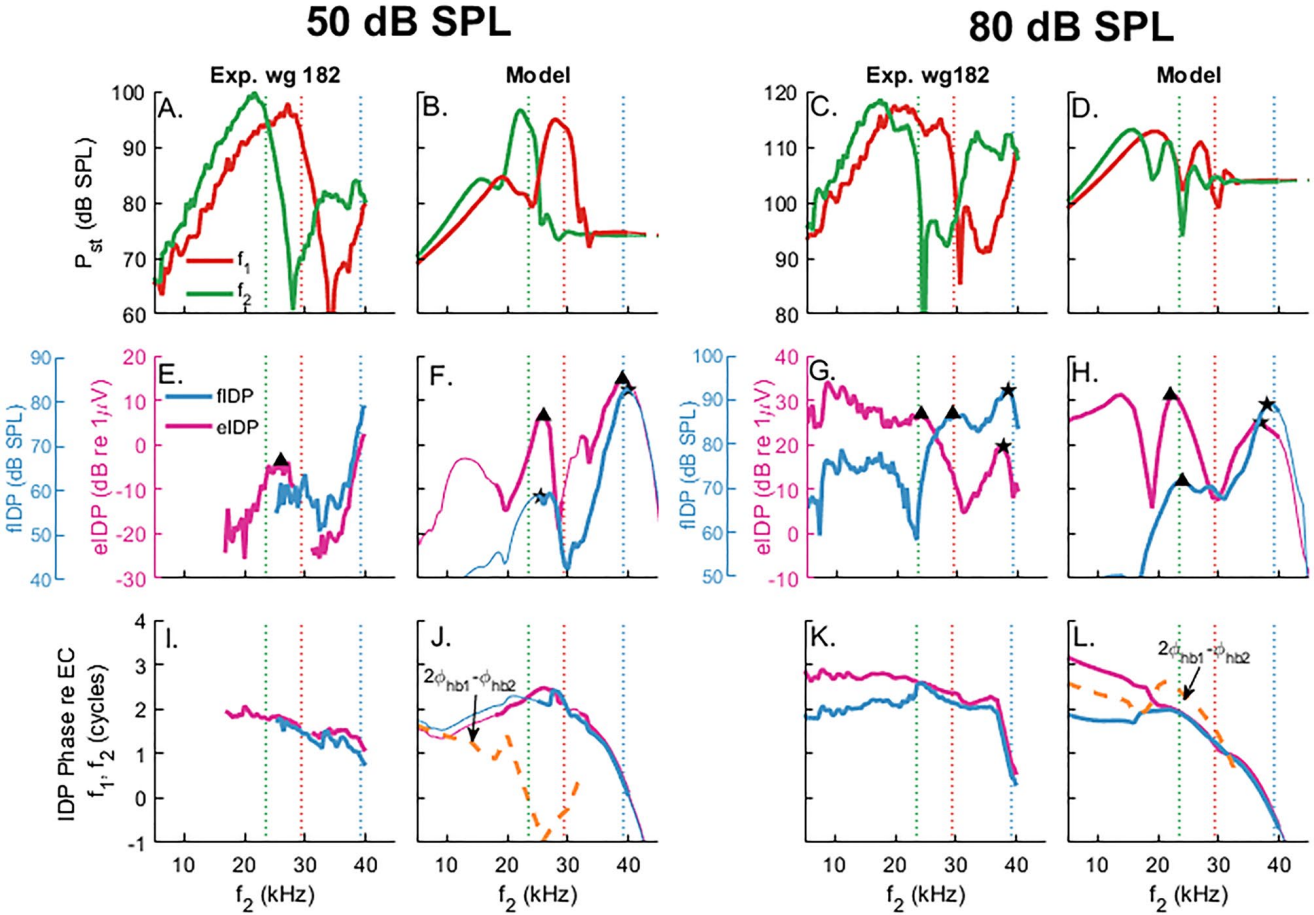
Mechanical and electrical two-tone responses of the cochlea: experiments (wg182) and model simulations in response to stimuli with equal primary levels of 50 dB SPL (**A,B,E,F,I,J**) and 80 dB SPL (**C,D,G,H,K–L**). The frequency ratio f_2_/f_1_ was constant (*f*_2_/*f*_1_ = 1.25). The figure includes both the response at the primary tone frequencies (**A–D**) and the IDP frequencies (**E–L**). All these components are plotted as a function of *f*_2_ rather than versus their own frequency so that key features of the IDP response can be easily related to where the primaries peak. (**A–D**) Shows the magnitude of the response of the primaries in the ST pressure (*f*_1_: thin red line; *f*_2_: thin green line). (**E–L**) Show the magnitude (**E–H**) and phase (**I–L**) IDP response in ST pressure and ST voltage. The phase in (**I–L**) is relative to $2{{\Phi }}_{{{\text{ec}}1}} - {{\Phi }}_{{{\text{ec}}2}}$, where ${{\Phi }}_{{{\text{ec}}1}}$ and ${{\Phi }}_{{{\text{ec}}2}}$ denote the phase of the primaries in the EC pressure. The green, red and blue vertical dotted lines correspond to the cases $f_{2} =$ BF, $f_{1} =$ BF, and $f_{{DP}} =$ BF, respectively. The thin lines in model simulations correspond to frequency range where no experimental data was collected. The yellow dashed lines in panels J and L correspond to $2\Phi _{{hb1}} - \Phi _{{hb2}}$, where $\Phi _{{hb1}}$ and $\Phi _{{hb2}}$ denote the phase of HB deflection for the *f*_1_ and *f*_2_ primaries, respectively.

Figure 3 extends these observations and shows the frequency-dependent coupling between mechanical and electrical responses to the IDP. *P*_*st*_ and LCM were obtained in response to two-tone stimuli with equal intensity primaries at frequencies *f*_1_ and *f*_2_ with a fixed *f*_2_/*f*_1_ ratio at 1.25. While IDPs and DPOAEs are generated at a family of different intermodulation frequencies, this manuscript focuses on the frequency component 2*f*_1_ − *f*_2_, which has the largest magnitude and is the most-studied DPOAE.

The top row of Fig. 3 shows the magnitudes of the *P*_*st*_ primaries plotted as a function of the *f*_2_ frequency. The *P*_*st*_ primaries correspond approximately to the input to the nonlinearity (direct in vivo measurements of HB deflection are not available from the experiments). The ST pressure responses at $f_{1}$ and $f_{2}$ (panel A–D) peak at frequencies lower than the BF of the measurement location because the traveling wave peaks at a lower frequency or more basal location for higher SPL (see Fig. 2 and also^22^). The relative smaller response at $f_{1}$ results from two-tone suppression effects. The experimentally observed notches in the $P_{{st}}$ amplitude are due to the interaction between compression and traveling waves and are duplicated in the model predictions.

The middle and bottom rows of Fig. 3 show the magnitude and phase of the IDPs in *P*_*st*_ (fIDP) and LCM (eIDP). At primary intensities of 50 dB SPL, the IDPs observed in both experiment and model simulation have two peaks corresponding to *f*_2_ either below or above the BF (Fig. 3E,F). A small peak (identified by triangles in panels E,F) is observed in the fIDP and eIDP within the so-called *primary overlap region,* where IDPs are expected to be locally generated by OHCs due to nonlinear distortion. After generation within this overlap region, the fIDP propagates in the forward direction. When the IDP frequency is close to the BF ($f_{{{\text{DP}}}} ~$ = 23.5 kHz when $f_{2} = 39.2~$ kHz), this forward propagating component (identified by a negative phase slope, see responses for $f_{2} > 30$ kHz in panels I,J) will stimulate OHCs, causing the second peak in the response (star symbol). This peak is only partially visible in panel E because the experimental data were not recorded beyond *f*_2_ = 40 kHz. With *f*_2_ < BF, the model predicts that the fIDP is dominated by wave propagating in the reverse direction from the overlap region towards the stapes (positive phase slope, see Fig. 3J) together with a drop in the magnitude of fIDP and eIDP. The experimental data of Fig. 3I does not seem to agree with the model predictions, as the eIDP phase seems to be dominated by forward wave propagation when *f*_2_ < BF. However, the frequency range over which the experimental data remains over the noise floor is quite limited in panel I, making it difficult to evaluate how IDPs propagate when *f*_2_ < BF.

The IDP responses to 80 dB SPL primaries share several similarities with the IDP responses to 50 dB SPL primaries. The key difference with the 50 dB SPL results is that the eIDP response is now low-pass, while the fIDP magnitude remains tuned with a global maximum when $f_{{{\text{DP}}}}$ is slightly below the BF (panel G). When $f_{{{\text{DP}}}} \sim BF$, both fIDP and eIDP have an amplitude peak (stars in panels G,H) with a negative slope in the phase responses (panels K,L), suggesting they are dominated by forward-propagating components. At *f*_2_ < BF, eIDP is low-pass and fIDP is band-pass, different from the quick drop off in $P_{{st}}$ at the primary frequencies. Although the interpretation of the phase can be somewhat tricky as a positive slope can appear in the absence of any reverse traveling components, the shallow positive phase slope for fIDP suggests dominance by reverse traveling components. However, the phase of the eIDP deviates from the phase of the fIDP (panels K,L).

The model was used to interpret the difference between the fIDP and eIDP phases at low *f*_2_ frequencies in response to 80 dB SPL primaries. The input to the nonlinearity, *i.e.* the stimulus to the transduction channels of the OHCs, is the HB deflection in the model. Figure 3L shows that the eIDP phase follows $2{{\Phi }}_{{{\text{hb}}1}} - {{\Phi }}_{{{\text{hb}}2}}$ for *f*_2_ < 20 kHz (where ${{\Phi }}_{{{\text{hb}}1}}$ and ${{\Phi }}_{{{\text{hb}}2}}$ denote the phase of HB deflection relative to the EC pressure for the *f*_1_ and *f*_2_ primaries, respectively). Because the $2f_{1}$ − $f_{2}$ eIDP arise from a cubic nonlinearity, this result implies that it is locally generated due to nonlinear distortion at low *f*_2_ frequencies in response to 80 dB SPL primaries.

Overall, the experimental observations and theoretical predictions suggest that the fIDP and eIDP are not as simple as the forward traveling primary responses. The measured IDPs include three different components: (1) a locally generated component that results from nonlinear distortion (2) a component traveling forward to its own BF place, and (3) a component that travels to the stapes in the reverse direction. Which component dominates can be recognized from the phase characteristics of the IDPs and corresponding *f*_2_ frequency. Also, how local DP generation depends on the local magnitude of the *f*_1_ and *f*_2_ components of the HB deflection contributes to the details of the observed/predicted IDP. For example, the deep notch in the eIDP at *f*_2_ ~ 19 kHz (panel H) arises because the *f*_2_ component of the HB deflection has a much larger magnitude than the *f*_1_ component (see the analysis in Fig. S5, and the experimental observations in wg165 in Fig. S4 in Supplementary Information, which exhibit a similar notch)”.

### eIDP arises from two different generation mechanisms

**Figure 4:**
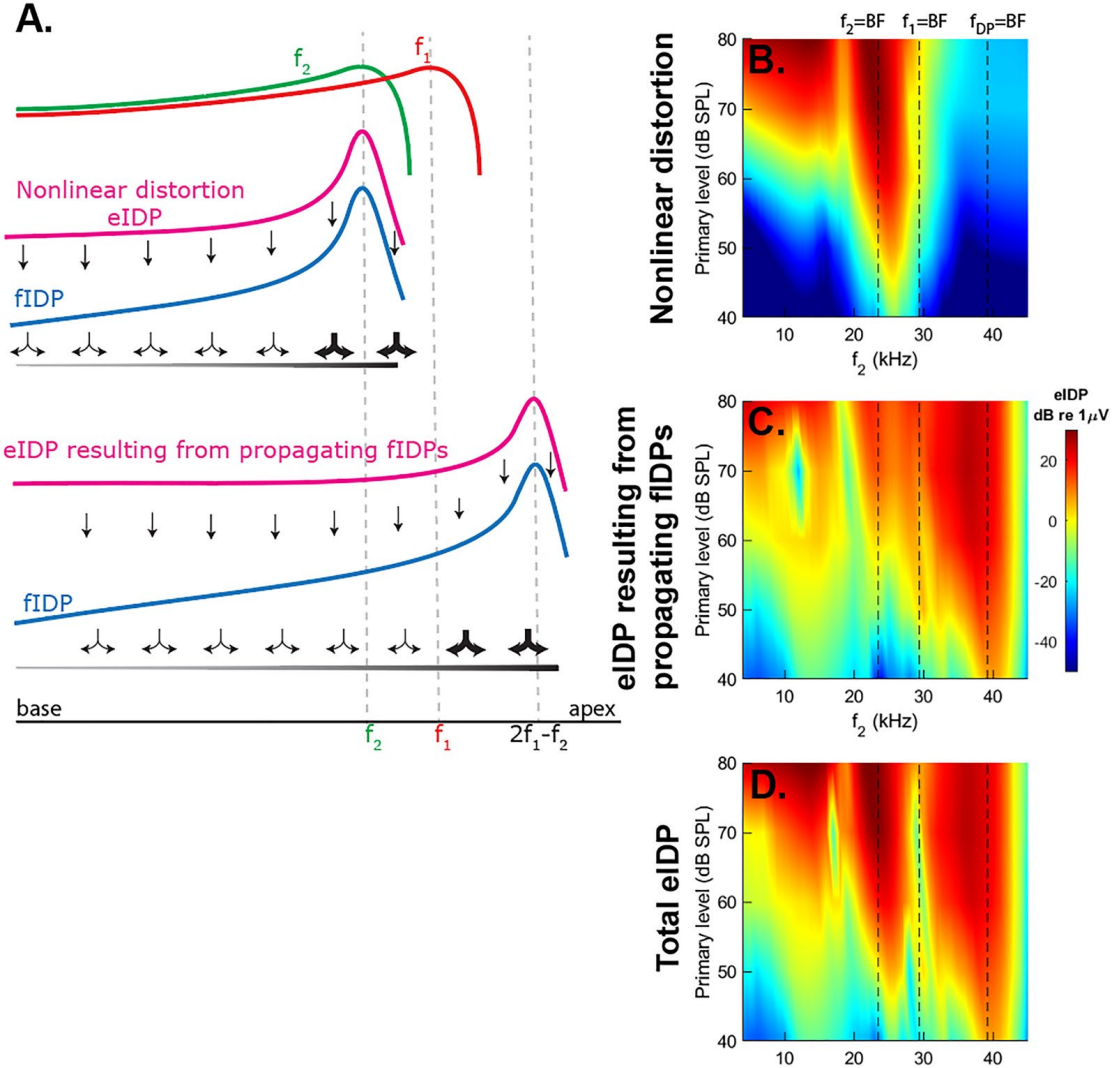
Generation mechanisms of eIDP and ear-canal DPOAEs. (**A**) Cartoon to illustrate the steps in generating an ear-canal DPOAE. A two-tone stimulus (with frequencies *f*_1_ and *f*_2_, *f*_2_ > *f*_1_) causes two traveling waves along the BM. At each location in the cochlea where these two tones cause vibrations, OHCs generate an electrical IDP due to local nonlinear distortion. The amplitude and phase of this local nonlinear distortion component is measured in the LCM (eIDP; magenta), and depends on the local stimulus properties. This electrical signal results in an OHC-generated force that can augment the BM vibrations and cause fluid pressure intracochlear DPs (fIDPs; blue), but only over a range where the BM impedance and mechanical load of the ST fluid are amenable. While propagating along the BM, these fIDPs also act as a stimulus for OHCs, forming a second, non-local source for eIDPs (the component resulting for propagating fIDPs) and fIDPs. The combination of all fIDPs that propagate back to the ear canal create the DPOAE that can be measured with a microphone. (**B–D**) show the decomposition of eIDP predicted by the model into local nonlinear distortion voltage and the voltage resulting from propagating fIDPs. The magnitude of eIDP is plotted as a function of $f_{2}$ and of the SPL of the primaries. (**B**) Shows the nonlinear distortion component, calculated using Eq. (S13). (**C**) Shows the eIDP resulting from propagating fIDPs, calculated using Eq. (S14). (**D**) Shows the total eIDP. The dotted lines indicate the value of the frequency *f*_2_ when *f*_2_, *f*_1_ or fDP is equal to the BF of the measurement location (23.5 kHz).

To understand how DPOAEs arise from IDPs consider the cascade of generation events illustrated in Fig. 4A. In response to a two-tone stimulus, the OHCs generate an eIDP due to *local nonlinear distortion*, which is the primary mechanism for eIDP generation. The eIDPs cause the OHC force to drive BM vibrations only over a spatially limited extent that is determined by the local impedance of the BM to create fIDP waves that propagate both in forward and reverse directions. These propagating fIDP waves stimulate OHC HBs and cause the generation of a mechano-electrical transduction current at the DP frequency. This second eIDP component will be called *the eIDP resulting from propagating fIDPs*. In contrast to the distortion component, which is generated due to the nonlinearity of the transduction channel, the eIDP resulting from propagating fIDPs relies on a quasilinear generation mechanism (because the magnitude of the IDP response is relatively small, even for 80 dB SPL primaries, nonlinear compression is nearly negligible in the generation of this secondary eIDP source). In similar vein, these secondary eIDPs may also create fIDPs that propagate towards the base of the cochlea. The primary and secondary fIDPs that propagate to the stapes combine to form the DPOAEs recorded in the ear canal.

Figure 4D shows model simulations for the total eIDP for primary levels between 40 and 80 dB SPL. In a manner consistent with the experimental observations of Fig. 3, the total eIDP increases with the primary level and shows multiple peaks. Induced by low intensity two-tone stimulus (< 60 dB SPL), the eIDP exhibits two peaks, one at $f_{2} \approx$ BF (in the overlap region), and one at $f_{{{\text{DP}}}} ~ \approx$ BF. At higher stimulus levels, the total eIDP response is maximum over a wide frequency range below BF (which is consistent with the low-pass nature of the eIDP response at high SPL), and has a local peak when $f_{{{\text{DP}}}} \approx$ BF.

We used the computational model to further explain the complex features of eIDPs and to decompose them into the nonlinear distortion contribution (Fig. 4B) and the eIDP resulting from propagating fIDPs (Fig. 4C) (see Supplementary Information for details on the calculations). With increase of primary levels, the local, nonlinear distortion eIDP not only peaks at $f_{2} ~\sim$ BF, but extends toward the sub-BF region, especially at high primary levels above 60 dB SPL (Fig. 4B). This is because of the transition of the primary responses from a band-pass to a low-pass filter shape (see Fig. 1E,F). When $f_{1}$ > BF, the eIDP is dominated by the component resulting from propagating fIDPs at all primary levels (Fig. 4C). In this region, this component comes from the forward propagating fIDP wave (see Fig. S6 in Supplementary Information) which is amplified by OHCs as it approaches its BF, causing a peak in both the component resulting from propagating fIDPs and the total eIDP. When $f_{2} \approx$ BF, the generation of the local, nonlinear distortion component is strongest and dominates the total eIDP response. In the sub-BF region, which component has the highest magnitude depends on the level of the primary tones. Below 65 dB SPL, the component resulting from propagating fIDPs that arises from the fIDP waves propagating in the reverse direction is the largest component (see also Fig. S4). Only at high primary levels is the local, nonlinear eIDP generation in response to the stimulus tones sufficient to dominate the total eIDP response.

### Correlation between DPOAE and IDP confirmed with local cochlear damage

**Figure 5:**
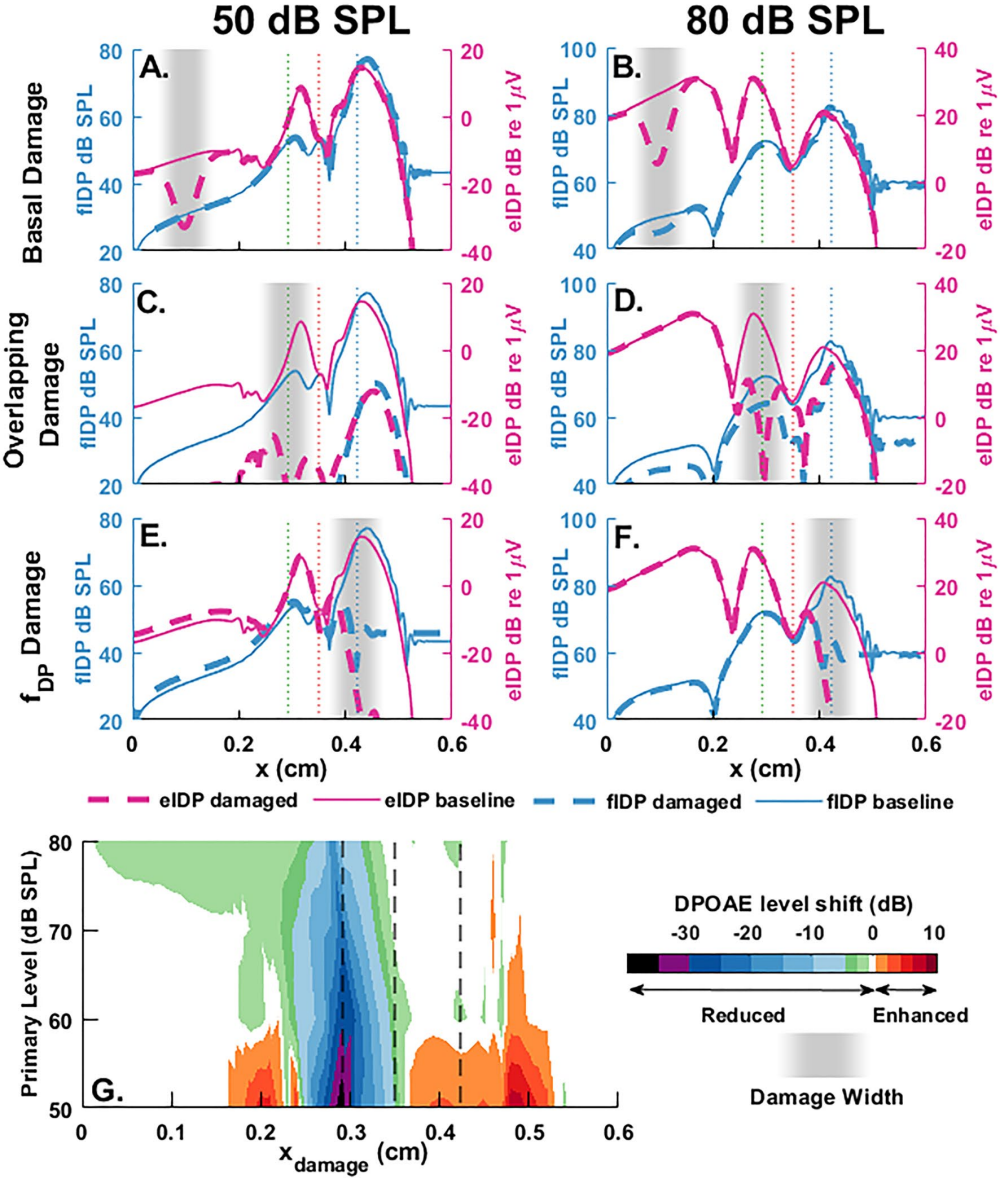
Effect of local OHC damage on the generation of IDPs and DPOAEs. (**A–F**) show the spatial responses of the eIDP (magenta lines) and fIDP (blue lines) for 50 dB SPL primaries (left panels) and 80 dB SPL primaries (right panels). The solid lines correspond to the baseline case; the dashed lines to the response when local damage is applied. The region of the damage shown in gray shaded area, includes approximately 20 sections of OHCs, and varies from a basal location (**A,B**), the overlap region (**C,D**) and the IDP tonotopic place (**E,F**). (**G**) Change of the DPOAE from its baseline value when the damage location is swept from the base to the apex for equal-level primaries of sound pressure levels between 50 and 80 dB. For all panels, $f_{2}$ = 20 kHz. In all panels, the vertical dashed lines correspond to the *f*_2_, *f*_1_ and *f*_DP_ tonotopic places.

The observation of significant eIDPs in the sub-BF region suggests that basal OHCs may contribute to the DPOAE measured in the EC, provided that the resulting OHC force is able to drive BM vibrations to create a (reversely propagating) fIDP wave. However, the ability of the OHC force to drive BM vibrations may be spatially restricted due to the BM impedance and load of the intracochlear fluids on the BM. To determine how different longitudinal locations give rise to fIDP waves and DPOAEs, we mimicked the effect of OHC damage by locally eliminating OHC mechano-electrical transduction in the physiologically motivated model (see details and Fig. S7 in Sect. 7 of the Supplementary Information). While the experimental data are measured at a fixed position and plotted versus *f*_2_, description of IDP generation versus longitudinal distance from the stapes is perhaps more intuitive. Figure 5 illustrates the modeled effects of local OHC damage on the IDPs and DPOAE induced by *f*_2_ = 20 kHz with *f*_2_/*f*_1_ = 1.25. Local damage effects on IDPs are shown for three different damage locations: a basal region (Fig. 5A,B), the primary overlap region (Fig. 5C,D), and the $f_{{{\text{DP}}}}$ region (Fig. 5E,F). The pre- and post-damage fIDP and eIDP responses are plotted for primary tones of levels 50 dB SPL (left column) and 80 dB SPL (right column). The change in the magnitude (in dB scale) of the DPOAE from its baseline value is plotted in Fig. 5G as the damage location is swept from base to apex for two-tone stimuli of levels between 50 and 80 dB SPL.

In response to 50 dB SPL primaries, OHC damage in a region basal to the *f*_2_ tonotopic place causes a ~ 20 dB local decrease in the eIDP within the damage region, but little change in the fIDP (Fig. 5A), and no change in the DPOAE (Fig. 5G). These observations can be explained by the facts that, at the base, the fIDP originates from more apical locations while the eIDP is locally generated. Because the fIDP is not strongly coupled to the basally generated eIDP, the OHC-generated eIDP contributes little to the DPOAE. For the case of 80 dB SPL primaries, the basal OHC damage causes local reductions of about 20 dB in eIDP and 5 dB in fIDP (Fig. 5B). The basal nonlinear distortion component, which, as demonstrated earlier, is the main source of basal eIDPs in response to high primary levels (Fig. 4), appears to have a small effect on basal fIDPs and DPOAEs (Fig. 5G).

Applying local damage in the primary overlap region has level-dependent effects on the IDP response (panels C,D). For 50 dB SPL primaries (Fig. 5C), the damage reduces eIDP and fIDP throughout the cochlea by a significant amount (> 30 dB) and the DPOAE by up to 38 dB SPL (Fig. 5G). These results strongly suggest that the DPOAE is primarily generated within the primary overlap region. At the higher primary level of 80 dB SPL (Fig. 5D), the reduction in the eIDP and fIDP at locations that are either close or apical to the damage region is smaller than for 50 dB SPL primaries. However, the basal eIDP, which is also generated due to local nonlinear distortion, is unaffected since the base is not within the damaged region, while both the basal fIDP and the DPOAE are reduced by around 10 dB.

Finally, the main effect of applying OHC damage around the $f_{{{\text{DP}}}}$ tonotopic place (Fig. 5E,F) is a pronounced reduction in the fIDP and eIDP in the peak region, which is caused by the elimination of the eIDP resulting from propagating fIDPs that propagates forwardly to its own BF place.

Overall, the damage has a progressively smaller effect on the DPOAE as the center of the damaged region moves away from the overlap region towards either the base or apex of the cochlea (Fig. 5G). As the SPL of the primaries is increased, the maximum reduction in the DPOAE level becomes smaller and the damage location that causes the maximum DPOAE reduction shifts slightly towards the base, because of the effect of stimulus levels on the peak locations of the response to the primaries. Damage causes a small but measurable reduction in the DPOAE evoked by high level stimuli (80 dB SPL) even when applied at locations significantly basal to the *f*_2_ tonotopic place (see the green area around $x_{{damage}}$ = 0.1 cm in panel G), which is consistent with the small reduction in the fIDP response observed in panel D. For low level primaries, two reddish regions, corresponding to an enhancement of the DPOAE, are observed about 0.1 cm basal to the *f*_2_ tonotopic place ($x_{{damage}} \approx 0.2$ cm) and near the $f_{{{\text{DP}}}}$ tonotopic place ($x_{{damage}} \approx 0.5$ cm). The enhancement observed when damage is applied basal to the *f*_2_ BF place is presumably because the damage eliminates cancellation from reverse propagating fIDP waves originating from a wide region. However, the enhancement observed when damage applied near the $f_{{{\text{DP}}}}$ tonotopic place (as well as small enhancement of the basal fIDP in Fig. 5E) is due to the introduction of a reflection mechanism for forward traveling waves when damage is applied around the BF region, as shown in an analysis included in the Supplementary Information (Fig. S8). Overall, the similarities between Figs. 4B, 5G (especially if we ignore the enhancement caused by the reflection introduced by the damage in Fig. 5G) are striking and show that OHC damage has a significant effect on the DPOAE only when applied in a region where the eIDP is dominated by local nonlinear distortion.

## Discussion

The experimental and modeling results of this paper clarify the regions and mechanisms of IDP generation by OHCs. We demonstrate for the first time that eIDPs measured in the LCM originate from a broader and more basally extended cochlear region than fIDPs measured in *P*_*st*_ or mechanical IDPs measured in the BM velocity. eIDPs are electrical potentials which arises from the nonlinearity of transduction channels in response to a two-tone stimulus, while the IDPs measured in the mechanical or fluid response require electromechanical conversion of the eIDPs into a mechanical response due to electromotility. Hence, the LCM can be considered to more directly reflect IDP generation by OHCs than *P*_*st*_ or the BM velocity. The low frequency eIDP response is related to the recent discovery of nonlinearity of the OHC, RL and TM responses at frequencies significantly below BF^4,23–25^. While both *P*_*st*_ and BM velocity respond linearly at sub-BF frequencies, the LCM exhibits a nonlinear response even at the lowest frequencies. The nonlinearity of the low frequency response of OHC and RL is not limited to the pure tone response but extends to the generation of IDPs in response to a two-tone stimulus.

The observation of eIDPs at sub-BF frequencies implies that OHCs generate an electromotile force at these low frequencies. In that frequency range, the force does not affect significantly BM vibrations at moderate SPLs but causes vibrations of the RL, according to recent data^16^ and to model simulations (see Fig. S1C in Supplementary Information). In the current model, these observations are due to the different impedance of the structures attached to the basolateral and apical ends of OHCs: the RL and TM are less stiff than the BM, which is as hypothesized in the literature^16,26^ (see also Figs. S2, S3 in Supplementary Information) and consistent with experimental estimates of the BM and TM^27,28^. Other recent cochlear models^29,30^ have also relied on the use of a stiff BM and a compliant RL to explain the different effects of OHCs on the vibrations of these structures. Because of the high stiffness of the BM, OHCs amplify BM vibrations only in a narrowband around BF, when the magnitude of the BM impedance drops due to the interplay between the BM stiffness and fluid inertia.

As in the case of the pure tone response^24^, the IDP responses predicted for the RL and LCM are very similar to each other but quite different from the IDPs observed in *P*_*st*_ or BM velocity. Noninvasive measurements of stimulus frequency OAEs in cochleae with perfused salicylate are also consistent with the notion that the RL is not directly coupled to the BM traveling waves^31^. The fact that eIDPs are reduced by basal OHC damage even though fIDPs are unaffected (for low-level primaries) is reminiscent of the recent observations of two-tone suppression of the RL response for probe tones below the BF^32^. Here, suppression has a similar effect as local damage because it causes saturation of the mechano-electrical transduction channels.

Both experimental observations and model predictions are consistent with two different mechanisms of eIDP generation by OHCs. The primary mechanism is *local nonlinear distortion*, which occurs predominantly in the overlap region where the mechanical responses to both primaries have a large magnitude. This region, which is narrow in response to low level primaries, broadens and extends toward the base as the level of the primary tones is increased because of the effect of stimulus level on the responses to the primary tones. Because of this, eIDPs are observed over an extended spatial region (when measured for a fixed *f*_2_ frequency) or a wide frequency range (when measured at a fixed location) in response to high level primaries. Because of electromotility, the local generation of an eIDP due to nonlinear distortion causes vibrations of the BM, which interacts with the intracochlear fluid, such that a fIDP is observed. However, the eIDP to fIDP conversion is spatially limited by the BM properties, such that the fIDP is present over a narrower frequency range than the eIDP. The secondary mechanism of eIDP generation is what we label the *component resulting from propagating fIDPs,* as it arises from the stimulation of the OHC HBs by the forward and reverse propagating fIDP waves that originate from the overlap region. At all SPLs, this component is the main source of eIDP in the DP BF region, where a peak is observed due to the amplification of the forward propagating fIDP wave. In response to low to moderate level (< 65 dB SPL) primaries, eIDPs at locations significantly basal to the peaks of the primaries are dominated by this secondary component.

OAEs are thought to arise from two different mechanisms: nonlinear distortion and coherent reflection^27^. These mechanisms relate to the origin of IDP wavelets that travel towards the cochlear base. The local nonlinear distortion mechanism we describe here is fundamentally the same as in that OAE-taxonomy. The coherent reflection mechanism is, however, different from our component resulting from propagating fIDPs. In the coherent reflection theory, it is hypothesized that forward propagating fIDP waves are reflected due to random perturbations in the impedance along the cochlear duct. These reverse fIDP wavelets interfere coherently when adjacent “reflectors” have the appropriate phase relation between them, which only occurs near the DP peak region, thus restricting the extent over which they contribute to DPOAEs. The secondary eIDPs (which happens throughout the cochlea) yields the subsequent spatially limited generation of the secondary fIDP components as we describe here. Previous literature^14,33^ has shown that the nonlinear distortion component tends to dominate in the EC at the relatively wide frequency ratio (*f*_2_/*f*_1_ = 1.25) used in the current study. Because the physiologically based model used in this study does not include cochlear roughness, forward propagating fIDP waves are not reflected, such that the eIDP resulting from propagating fIDPs does not generate reverse propagating waves in the BF region. However, if cochlear roughness was introduced such that forward propagating fIDP waves are reflected, the contributions from the eIDP resulting from fIDP propagating waves might be enhanced at the base of the cochlea and in the EC, particularly at lower frequency ratios. For the wide ratio used in this study, our simultaneous measurements of eIDPs and fIDPs and simulations without a reflection component demonstrate that eIDPs originating from the nonlinear distortion region are the primary intracochlear source of DPOAEs.

Simulating the effect of local OHC damage (Fig. 5) allowed us to probe the relations between the two mechanisms of eIDP generation and their influence on DPOAEs measured in the EC. Simulation results are consistent with experimental observations from Dong and Olson^34^, who characterized the effect of local damage on fIDPs and DPOAEs evoked by high intensity primary tones. As in the current study, for *f*_2_/*f*_1_ = 1.25, Dong and Olson observed a correlation in the reductions of DPOAEs and fIDPs when damage occurred at a region of *f*_2_ frequency close to the BF, but not when *f*_DP_ was close to the BF. The near complete elimination of the eIDPs when damage is applied in the overlap region implies that the observation of eIDPs or fIDPs at any cochlear location requires local nonlinear distortion generated by healthy OHCs in this overlap region. At low to moderate SPL, a reverse propagating fIDP wave that originates from the overlap region is measured at the base of the model representative of the undamaged cochlea. However, applying damage in a basal region causes a significant reduction in the eIDP, because the eIDP measured at the base is dominated by the component resulting from propagating fIDPs, which requires mechano-electrical transduction at the measurement location. These results imply that the basally generated eIDP component resulting from propagating fIDPs has limited influence on the fIDPs. Applying local damage in the DP BF region eliminates the OHC electromechanical feedback necessary of the amplification of the IDP response, which significantly reduces the magnitude of both the fIDP and eIDP. Hence, a normal IDP response in the BF region and normal perception of IDP vibrations require healthy OHCs in both the overlap and DP BF regions.

Whether DPOAEs include contributions from regions located significantly basal to the *f*_*2*_ tonotopic place is an important debate in OAE literature, with both scientific and clinical implications. Precise understanding of which cochlear regions contribute to DPOAEs is essential for the diagnosis of frequency-dependent sensorineural hearing loss. We have shown that IDPs are generated by OHCs primarily at the peak locations of the responses to the primary tones, and also at locations significantly closer to the cochlear base (see also^14^). This is especially true in response to high-level primaries (which are often required to measure DPOAEs for clinical diagnosis, especially for subjects with some level of sensorineural hearing loss). Model simulations with local OHC damage show that DPOAEs primarily originate from locations close to the *f*_2_ tonotopic place in the healthy cochlea at low to moderate stimulus levels (up to 65 dB SPL). However, DPOAEs generated in response to high-level primaries (above 65 dB SPL) are found to include small contributions from basal IDP generators, which is consistent with some experiments which have used a high level suppressor tone to conclude that some of the DPOAEs originates from cochlear locations basal to the primary overlap region in humans^35^ and rabbits^36^. A recent cochlear model with a wide region of RL nonlinearity also found that the low frequency RL response has a limited influence on DPOAEs^29^. However, only the BM is directly coupled to the intracochlear fluid in the current model, as in most classical models of the cochlea. Basal IDP generators might contribute more significantly to DPOAEs in potentially more realistic cochlear models with direct coupling of the SM fluid to the RL and/or TM (such as^37,38^). Micromechanical measurements of the IDP response combined with simulations with high fidelity computational models will be needed to further understand the complex link between IDPs and DPOAEs.

## Methods

### Experimental methods

#### Animal preparation

All procedures involving experimental animals were approved by the Institutional Animal Care and Use Committees (IACUCs) of Columbia University and the VA Loma Linda Healthcare System that in accordance with relevant guidelines and regulations of Association for the Assessment and Accreditation of Laboratory Animal Care International (AAALAC). The study was carried out in compliance with the ARRIVE guidelines. Animal preparation and acoustic stimulation was as described in (Dong and Olson^4,39^). Experiments were performed in anesthetized young adult gerbils. Gerbils were anesthetized with ketamine (80 mg/kg) and xylazine (10 mg/kg), with maintenance doses (1/3 to ½ of initial dose) given as needed throughout the experiment, and the analgesic buprenorphine was administered every 6 h. At the end of the experiment the animal was euthanized with Pentobarbital. While anesthetized, animal body temperature was maintained at ~ 37° using a rectal probe attached to a Harvard Apparatus heating pad. The dorsal surface of the skull was fixed to a head-holder with dental cement. A tracheotomy was performed to maintain a patent airway. The left pinna was surgically removed and the bulla was widely opened. A small hole (diameter ~ 200 μm) was hand-drilled through the bony wall of scala tympani (ST) at the turn one location with best frequency (BF) around 20 kHz. A total of eleven animals were used in the study and five animals provided a complete dataset with consistent results. Main findings were illustrated by data from two representative cochleae under healthy conditions, shown in the main article and in Supplementary Information (Fig. S4).

Cochlear condition was assessed at key time points using compound action potential (CAP) thresholds measured at the round window using a silver-ball electrode, with the reference electrode connected to neck muscle having a threshold criterion of ~ 5 μV (p-p)^14,40^. In addition, DPOAEs and single-tone responses at the level of the BM were used to estimate the degree of nonlinearity of cochlear responses, which become linear if the preparation deteriorates.

#### Dual-sensor

The dual-sensor, described in detail in our previous publication (^4^), combined a custom-built pressure sensor (ID/OD: 75/125 μm) and an isonel-insulated platinum wire electrode (OD: 28 μm AM Systems, Sequim WA) side by side. The latter was cut to be approximately flush with the pressure sensor tip. Both the pressure sensor and electrode (with their associated amplifiers) are broadband with mild low-pass filtering over the frequency range of interest. The pressure sensor was calibrated in air at both room- and body-temperature and in water after construction, and before and after the experiment. Only when calibration results between air and water were within 3 dB was the sensor used in the experiment. The impedance of the electrode is ~ 1 M${{\Omega }}$ measured at 1 kHz. The wire electrode’s frequency response was characterized as described in Baden-Kristensen and Weiss^41^. At 40 kHz, the test electrode showed ~ 2 dB amplitude attenuation and phase roll off of ~ 30°. The dual-sensor was introduced into the cochlea through the ST hole, and was aimed at the sensory tissue (Fig. 1A). A silver reference electrode was placed in the neck. The dual-sensor was slowly advanced in micrometer steps with a motorized micromanipulator until contacting the BM, then retracted ~ 10 μm where responses were measured.

#### Sound stimulation and data acquisition

Single- or two-tone stimuli were generated by a Tucker Davis Technology (TDT) (Alachua, FL) system III (sampling frequency of 200 kHz) and delivered to the ear canal (EC) via a closed acoustical system that consisted of one or two electrically shielded Radioshack (Fort Worth, TX) tweeters. Synchronization of the three data acquisition channels (ear canal pressure, and the dual sensor output) was checked and the third channel’s relative delay (< 5 μs) was accounted for in the analysis. Stimulus and acquisition programs were written in MATLAB (The MathWorks, Natick, MA) and the TDT visual design studio. Responses waveforms were measured for ~ 1 s and time locked averaging was performed; the averaged data were stored in segments of 4096 points. The waveforms were later analyzed by Fourier transform with MATLAB. Sound pressure levels are reported as dB SPL (decibels relative to 20 μPa peak). The SPL was calibrated within 3 mm of the eardrum using an ultrasound Sokolich probe tube microphone, which has flat frequency responses up to 50 kHz.

### Physiologically motivated model of the cochlea

The computational model of the gerbil cochlea (Fig. 1B) used in this work has been described in a series of papers^20,21,33^. The model is based on the finite element method and includes acoustic (fluid), mechanical, and electrical physics. The model parameters were selected so that the model responses to a pure-tone stimulus are representative of measurements taken in the mammalian species used in the experiments, the gerbil. To tune the mechanical and electrical response to a pure tone, some changes were made to the electrical model and in the parameters values from the values listed for our most recent gerbil model^21^. The parameter values of the current model can be found in the Supporting Material. DPOAEs are commonly assumed to be generated due to nonlinear distortion from the overlap region and reflection by inhomogeneities on the cochlear partition close to the DP tonotopic place^42^. However, experimental data in the gerbil data suggest that, for the relatively wide primary frequency ratio of 1.25 used in this study, the DPOAEs are dominated by contributions from the distortion source^14^. Hence, the model used here does not include any cochlear roughness.

#### Fluid model

The cochlea is modeled as a rectangular box with two ducts (the ST and SV) filled with a fluid which is modeled as incompressible and inviscid (Fig. 1B). A three-dimensional (3D) model is used to represent the highly 3D response of the fluid response, particularly in the BF region^20,43,44^.

#### Organ of Corti complex model

A key component of the cochlear model is a model of the organ of Corti geometry and micromechanics, which includes one degree of freedom for the transverse displacement of the BM and degrees of freedom for the displacement of the TM in bending (transverse) and shear (radial) directions of the HB (Fig. 1B). The displacement of the BM is coupled to the intracochlear fluid in the ST and SV while the TM and RL are not directly interacting with the intracochlear fluid. As in^45^, longitudinal coupling is included for both the BM and TM mechanics.

#### Model of OHC mechano-electrical transduction and somatic electromotility

Deflection of the OHC HBs stimulates the mechano-electrical transduction channels. The mechano-electrical transduction current, $i_{{MET}}$, is the following nonlinear function of the HB deflection relative to the RL, $u_{{hb/rl}}$:

1 $$i_{{MET}} \left( {u_{{hb/rl}} } \right) = G_{{hb}}^{{\max }} \Delta V_{{hb}}^{0} \left( {\frac{1}{{1 + \exp \left[ { - \frac{{u_{{hb/rl}} - X_{0} }}{{\Delta X}}} \right]}} - P_{0}^{s} } \right),$$

where $G_{{hb}}^{{max}}$ is the saturating conductance, $\Delta V_{{hb}}^{0}$ is the resting value of the difference between the SM potential and intracellular OHC potential, $P_{0}^{s}$ is the resting open probability of the MET channel and $X_{0}$ and $\Delta X$ are constant displacements. The transduction current depolarizes the OHC main body. Electromotility is modeled using reciprocal linearized equations that relate the OHC electromotile force, $f_{{ohc}}$, the current, $i_{{som}}$, the OHC change of length (compression), $u_{{ohc}}^{{comp}}$, and the OHC transmembrane potential, $\Delta V_{{ohc}}$:

2 $$i_{{som}} = - \varepsilon _{3} \dot{u}_{{ohc}}^{{comp}} ,$$

3 $$f_{{ohc}} = \varepsilon _{3} \Delta V_{{ohc}} ,$$

where $\varepsilon _{3}$ is the electromechanical coupling coefficient of the OHC.

#### Electrical model of the scalae and OHCs

The electrical model includes degrees of freedom at each cross-section for the electrical potentials in the SV, SM, OHC and ST. As in^46^, the model includes longitudinal electrical cables to account for the longitudinal spreading of electrical currents in the cochlear ducts (see Fig. 1B). Due to the high computational cost of running simulations with the cables, these cables were not included in previous time-domain implementations of the model^20,21,47^. We found in this that electrical longitudinal cables (particularly in the ST) are essential for matching with a single set of model parameters the relatively high magnitude of the ST voltage at low frequency and in the BF region measured in response to a pure tone. The value of the space constant due to ST cable (134 μm) is in line with recent reports (80 μm in the data from^4^) and much lower than older estimates (0.5 to 4 mm in Ref.^48^).

#### Coupling of the cochlear model to the middle ear

The cochlear model is coupled to a 1 DOF middle ear model with parameters were chosen so that the middle ear has a relatively constant stapes reflectance when coupled to the cochlear model (see Fig. S9 in Supplementary Information). Based on experimental data in the gerbil^40^, the middle ear pressure transfer functions are assumed to have frequency-independent values (a gain of 30 dB and a constant delay of 32 µs in the forward direction; a gain of − 35 dB and a delay of 38 µs in the reverse direction).

#### Numerical methods

The partial differential equations that govern the nonlinear dynamics of the cochlear model are discretized using the finite element method. The resulting set of equation is formulated as a system of nonlinear ordinary differential equations (ODEs) using a state-state formulation^47,49^, which is solved in the time-domain using a Runge–Kutta ODE solver (*ode45* function in MATLAB). The frequency components of the response of the model are extracted from the time-domain response by applying a Fast Fourier Transform algorithm to the steady-state portion of the response.

## Supplementary Information

Supplementary Information.

## Data Availability

Experimental and simulation data are available from the corresponding authors.
