# Supplementary material for: Intracochlear distortion products are broadly generated by outer hair cells but their contributions to otoacoustic emissions are spatially restricted
Source: Sci Rep. 2021 Jul 1;11:13651. doi: 10.1038/s41598-021-93099-7 (PMC8249639; doi:10.1038/s41598-021-93099-7)
Supplement: Supplementary file 1 — Supplementary Information. [file 41598_2021_93099_MOESM1_ESM.pdf]

Supplemental information: Intracochlear distortion products are broadly generated by outer hair cells but their contributions to otoacoustic emissions are spatially restricted.

Authors: Thomas Bowling, Haiqi Wen, Sebastiaan W.F. Meenderink, Wei Dong, Julien Meaud

1. Mechanical power delivered by OHCs to the BM: The power delivered by OHCs to the BM in response to a pure tone is calculated using the equation:

$$\pi_{OHC/BM}(x,\omega) = \frac{1}{2} Re[f_{ohc}(x,\omega)(v_{BM}(x,\omega))^*], \qquad (S1)$$

where f_{ohc} and v_{BM} are the stimulus frequency component of the electromotile force (calculated using Eq. (3)) and BM velocity, and the subscript $*$ denotes the complex conjugate.

2. Electrical and micromechanical response of the model

While results in the manuscript focus on predictions of the LCM for direct comparison with the experiments, the electromotile OHC force depend on the transmembrane potential, ΔV_{ohc} (See Eq. 3 and Fig. 1B). Fig. S1A shows the magnitude of the transmembrane potential, ΔV_{ohc}, and the LCM relative to the EC pressure in response to a pure tone. ΔV_{ohc} has a more tuned response than the LCM. The magnitude of the transmembrane potential is predicted to be considerably higher than LCM, due to the low value of the resistance of the ST to ground, R_{tl} relative to the basolateral resistance of the OHC, R_m. These predictions are in line with experimental estimates, where the intracellular potential has been reported to be about 50 dB higher than the extracellular potential [1, 2].

The results of Fig. S1B show that the predictions of the relative broad response of LCM to low level stimuli and a low pass response of V_{st} to high level stimuli are due to the longitudinal electrical cable in the ST (see Fig. 1B). Based on the schematics of the electrical model in Fig. 1B, the equation for the LCM can be written as:

$$V_{st}(x,t) = \frac{1}{2\sqrt{r_{st}R_{tl}}} \int_0^L e^{-\frac{|x-\bar{x}|}{\sigma_{st}}} i_{ohc}(\bar{x},t)\, d\bar{x} \qquad (S2)$$

Where $i_{ohc}(x,t) = i_{MET}\big(u_{hb/rl}(x,t)\big) + \frac{1}{R_a}(V_{sm}(x,t) - V_{ohc}(x,t)) + C_a \frac{\partial (V_{sm}(x,t) - V_{ohc}(x,t))}{\partial t}$, r_{st} is the resistance per unit length of the ST cable, R_{tl} is the resistance of the ST to ground, and σ_{st} is the space constant for the ST, which is given by:

$$\sigma_{st} = \sqrt{R_{tl}/r_{st}} \qquad (S3)$$

If the longitudinal cable in the ST is neglected, LCM can be calculated from the value of the electrical current flowing through the OHC, i_{ohc}, using the equation $V_{st}(x,t) = R_{tl} i_{ohc}(x,t)$ The predictions, shown in black lines, from this approximate model that neglects the longitudinal cable in the ST, match nearly exactly the simulations of the model with the cable at low frequencies, when the wavelength of the traveling wave, λ_{TW}, is much larger than the space constant of the electrical longitudinal cables σ_{st}. However, in the BF region, λ_{TW} is much shorter, such that the electrical cables couple longitudinal sections that have electrical current terms, i_{ohc}, that are out of phase. Because of this, the model that

neglects the cable overestimates the magnitude of LCM in the BF region. This implies that the low-pass nature of LCM in response to high level SPL is primarily due to the cancellation caused by the electrical coupling between neighboring longitudinal locations.

The electromotile OHC force acts both on the BM and on the RL. Fig. S1C shows that the model captures some of the recent experimental observations regarding the nonlinear response of the RL to a pure tone [3-5]: the RL response is predicted to be nonlinear both in the BF region and at low frequency, as observed in recent experiments; at BF, the RL response to low level stimuli is about 10 dB higher than the BM response. These results demonstrate that the OHC force generation in the low frequency sub-BF region can influence the RL vibrations even through it does not influence the BM vibrations.

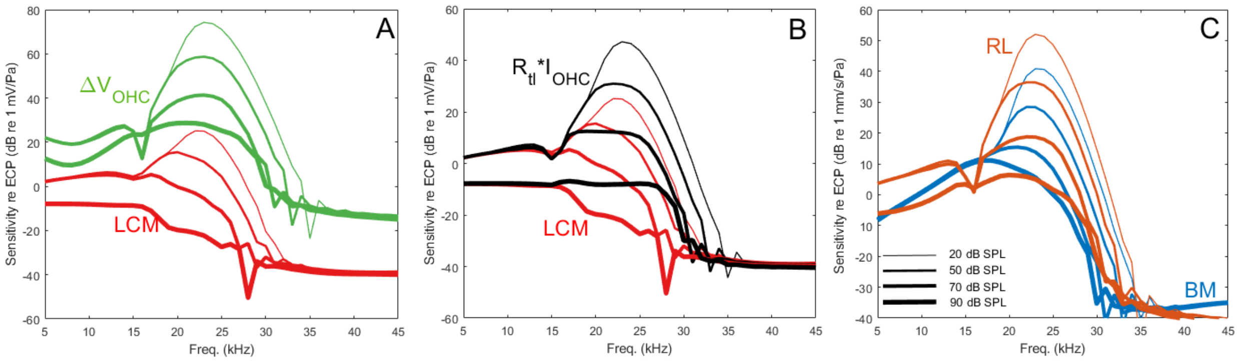

Figure S1. Response of the model to a pure tone of levels 20, 50, 70, and 90 dB SPL. A. Magnitudes of the ST potential and OHC transmembrane potential, relative to the EC pressure. B. Magnitude of V_{st} response relative to EC pressure in the model that includes electrical longitudinal cables (red lines) and in an approximate model that neglects the cables (black lines). C. Magnitude of BM velocity and RL transverse velocity relative to EC pressure.

3. Comparison of passive forces to OHC forces

The results of this paper and previous experimental results suggest the OHCs generate an electromotile force over a broad frequency range. However, the effect of this force on the BM vibrations is limited to frequencies around BF, while it can influence the transverse vibrations of the RL and TM even at low frequencies [3, 5].The model simulations were used to explain the differences between the effects of the OHC force on the BM and on RL/TM.

Because the transverse vibrations of the RL and TM are similar in the current model, the computational model was used to calculate the forces applied by the OHCs on the BM and TM compared to the other forces applied on the structures. The BM has a stiffness, damping and mass and, in addition to the electromotile force, is subject to passive forces due to the fluid and to interactions with the organ of Corti. Similarly, the TM has stiffness, damping and mass and is subject to passive mechanical forces from the organ of Corti and the electromotile force. The equations of motion for the BM and TM bending mode (TMB) can be written in the following forms:

$$Z_{bm}^{pass} v_{bm} = F_{f/bm} + F_{ohc/bm}^{act} + F_{OoC/bm}^{pass} \quad \text{(S4)}$$

$$Z_{tmb}^{pass} v_{tmb} = F_{ohc/tmb}^{act} + F_{OoC/tmb}^{pass}, \quad \text{(S5)}$$

where Z_{bm}^{pass} is the passive impedance of the BM, when uncoupled from the intracochlear fluid and organ of Corti (i.e., the contributions from the intrinsic stiffness, damping and mass of BM); Z_{tmb}^{pass} is the passive impedance of the TM bending (transverse) mode (due to the stiffness and damping of the TM attachment to the spiral limbus, the longitudinal viscoelasticity of the TM, and TM mass); $F_{ohc/bm}^{act}$ and $F_{ohc/tmb}^{act}$ are the active OHC force applied on the BM and TM; $F_{OoC/bm}^{pass}$ and $F_{OoC/tmb}^{pass}$ are the passive forces (i.e., due to the stiffness of OoC structures) applied by the organ of Corti components (OHC, RL, HB) on the BM and TM bending mode, respectively; $F_{f/bm}$ is the force applied by the fluid on the BM.

Fig. S2 shows the magnitude of the different terms of these equations in response to a pure tone. Because of the high stiffness of the BM, the OHC force is much smaller than the term $Z_{bm}^{pass} v_{bm}$ except around BF in response to low SPL stimuli. Hence, the OHC force has limited effect on the BM vibrations except around the BF region. For the TM, the OHC force is significant relative to $Z_{tmb}^{pass} v_{tmb}$ at all SPLs and throughout the frequency range. This is due to the fact that the TM has much lower stiffness than the BM (K_{tmb1}/K_{bm}=0.02 in the current model, see Table S1), such that the impedance of the TM is much lower than the impedance of the BM. Hence the OHC can influence the vibrations of the TM throughout the frequency range in response to a pure tone.

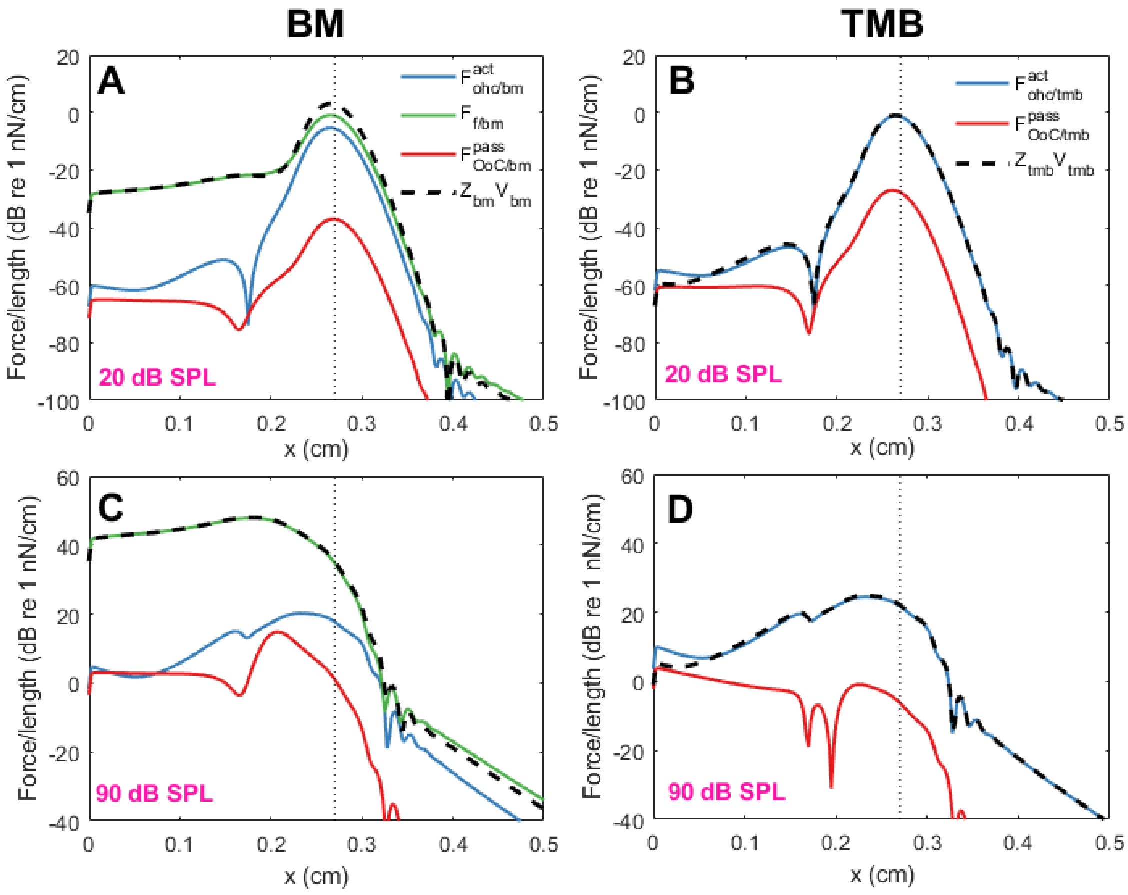

Figure S2. Forces per unit length applied on the BM (panels A and C) and on the TM bending mode (panel B and D), in response to pure tones of frequency 22 kHz and levels 20 dB SPL (A-B) and 90 dB SPL (C-D). The forces applied on the BM and TM are compared to the contributions from the impedance of the BM and TM, $Z_{bm}^{pass} v_{bm}$ and $Z_{tmb}^{pass} v_{tmb}$, shown in black dashed lines. The forces applied on the BM include contributions from the fluid (green lines), the coupling to organ of Corti structures (red

lines), and from the electromotile OHC force (blue lines). The forces applied on the TM bending mode include contributions from the coupling to organ of Corti structures (red lines) and from the electromotile OHC force (blue lines).

Similar results are found at the DP frequency in response to a two-tone stimulus (Fig. S3). As in response to a pure tone, the electromotile OHC force has a more significant effect on the TM bending mode vibrations at the DP vibrations than on the BM vibrations, due to the much lower stiffness of the TM bending mode than the stiffness of the BM.

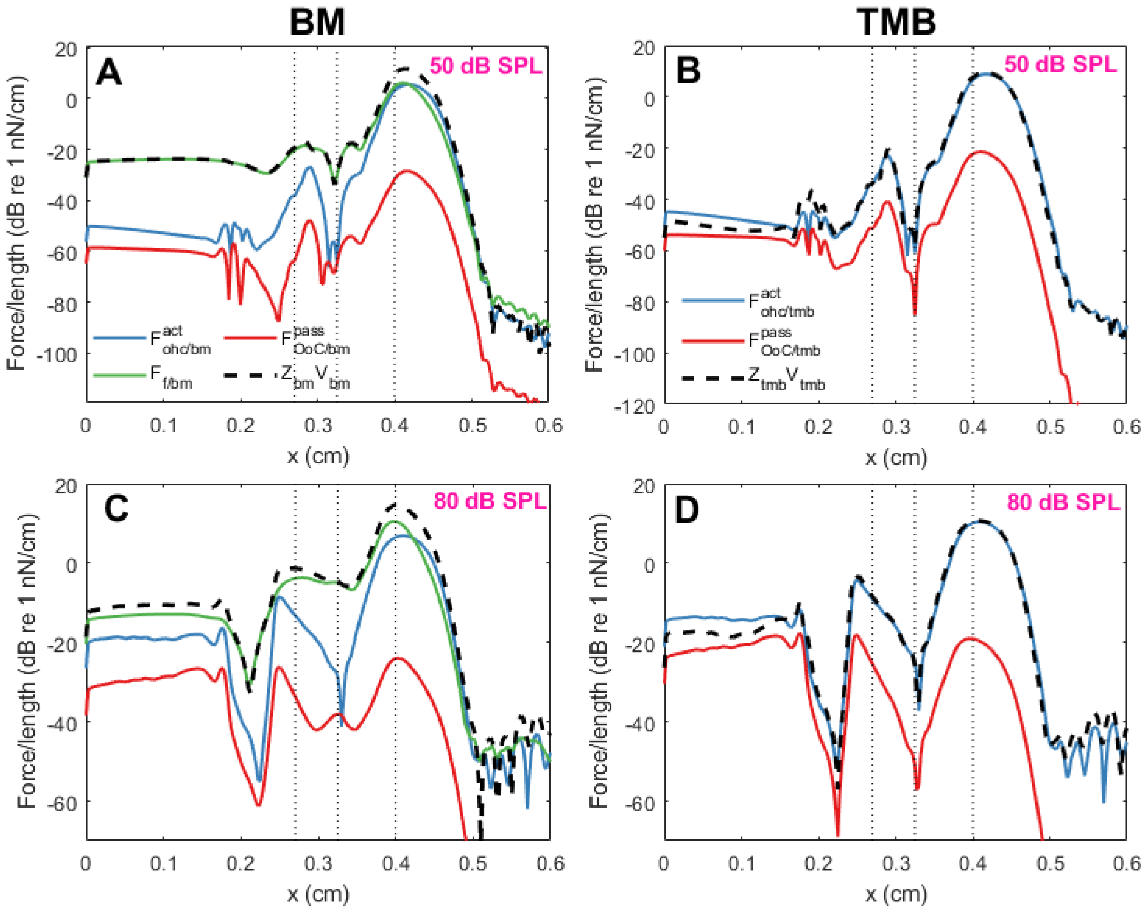

Figure S3. Forces per unit length applied on the BM (panels A and C) and on the TM bending mode (panel B and D) at the DP frequency in response to two tone stimulus of levels 50 dB SPL (A-B) and 80 dB SPL (C-D). The DP frequency is 22 kHz. The forces applied on the BM and TM are compared to the contributions from the impedance of the BM and TM, $Z_{bm}^{pass}v_{bm}$ and $Z_{tmb}^{pass}v_{tmb}$, shown in black dashed lines. The BM includes contributions from the fluid (blue line), the coupling to organ of Corti structures (red lines), and from the electromotile OHC force (blue lines). The TM includes contributions from the coupling to organ of Corti structures (red lines), and from the electromotile OHC force (blue lines)

4. IDP response: comparison of two experimental data sets and of model simulations

The influence of the SPLs and frequencies of the primary tones on the IDP is more systematically shown in Fig. S4, where the magnitude of IDP response is plotted for two different animal experiments (the same animal experiment as in previous figures, wg182, as well as animal experiment wg165) and for the model simulations. Several differences between the ST pressure (1st row of Fig. S4) and ST voltage (2nd row of Fig. S4) responses are apparent, despite some variability between the different data sets. In the model simulations and both of these experiments, the voltage response extends to low frequencies (especially in response to high level primaries), while the pressure response tends to be mostly limited to f_2 frequencies where the primaries and the DP are close to the BF of the measurement location. Interestingly, Fig. S4E. shows a notch at f_2 ≈15 kHz for low stimulus, as predicted by the model at a somewhat higher frequency (f_2 ≈19 kHz).

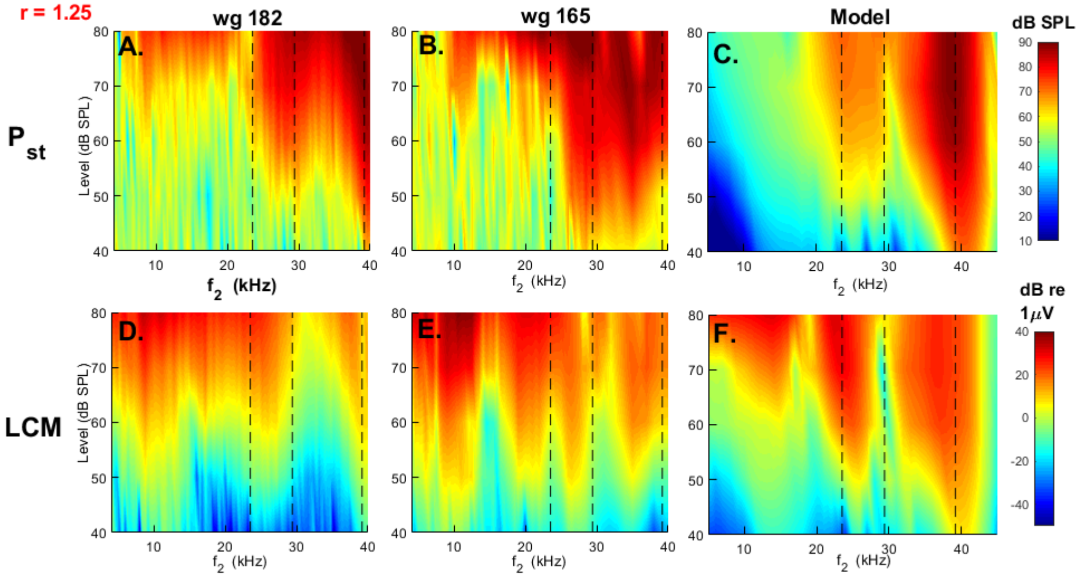

Figure S4. Influence of the SPL of the primaries on the generation of IDP in ST pressure and ST voltage. The frequency f_2 is swept (horizontal axis) while the frequency ratio is kept at 1.25; the level of the primaries is varied from 40 dB to 80 dB SPL (vertical axis). The figures show the magnitude of the IDP in the ST pressure (top panels) and in the ST voltage (bottom panel Results are shown for two different animal experiments (wg182 in A and D; wg165 in B and E) and model simulation (C and F). The vertical dotted lines indicate the value of the frequency f_2 when f_2, f_1 and f_{DP}, respectively, is equal to the BF of the measurement location (23.5 kHz). The notch in the model response around 19 kHz (panel F) and around 15 kHz in panel E are consequence of two-tone suppression.

5. Cause of the notch in the 80 dB eIDP

A notch is observed in the eIDP when f_2=19 kHz in response to 80 dB SPL primaries (Fig. 3H). The model was analyzed to explain the origin of this notch. The input to the nonlinearity in the model is the HB deflection (see Eq. 1). The levels of the f_1 and f_2 components of HB deflection predicted by the model for 80 dB SPL primaries is plotted in Fig. S5A. Using these inputs, the distortion product in the transduction current directly due to local nonlinear distortion (calculating using Eq. 1) is plotted as a function of f_2 in Fig. S5B. A similar notch predicted in the LCM in Fig. 3H is observed in Fig. S5B, implying that the notch in the eIDP originates from the specific form of nonlinear equation used in Eq. 1.

The DP in the transduction current only depends on the local values of the HB deflection. To understand the origin of the notch, the level of the HB deflection at the f_1 and f_2 frequencies was varied systematically, and the DP in the transduction current was calculated and plotted as a function of these levels in the surface plot of Fig. S5C. It is observed that the DP in the transduction current tends to be maximum when the level of the transduction current at the f_1 and f_2 frequencies are similar (the dotted line in Fig. S5 corresponds to the equi-level contour). The black solid line corresponds to the levels of the HB deflection at the f_1 and f_2 frequencies in response to 80 dB SPL primaries. For f_2=10 kHz and f_2=22 kHz (which corresponds to the diamond and square symbols, respectively), the levels of the two primaries on the HB deflection are nearly identical, which results in nearly maximum DP generation. However, for f_2=19 kHz (circle symbol in Fig. S5), the f_2 component of the HB deflection is significantly higher than the f_1 component, such that DP generation is significantly reduced. This explains why a notch is observed in the DP predicted in the transduction current or in the LCM when f_2=19 kHz. Because the fIDP is not generated locally at 19 kHz since it is dominated by reverse propagating waves, a notch is not observed at the same frequency in the fIDP.

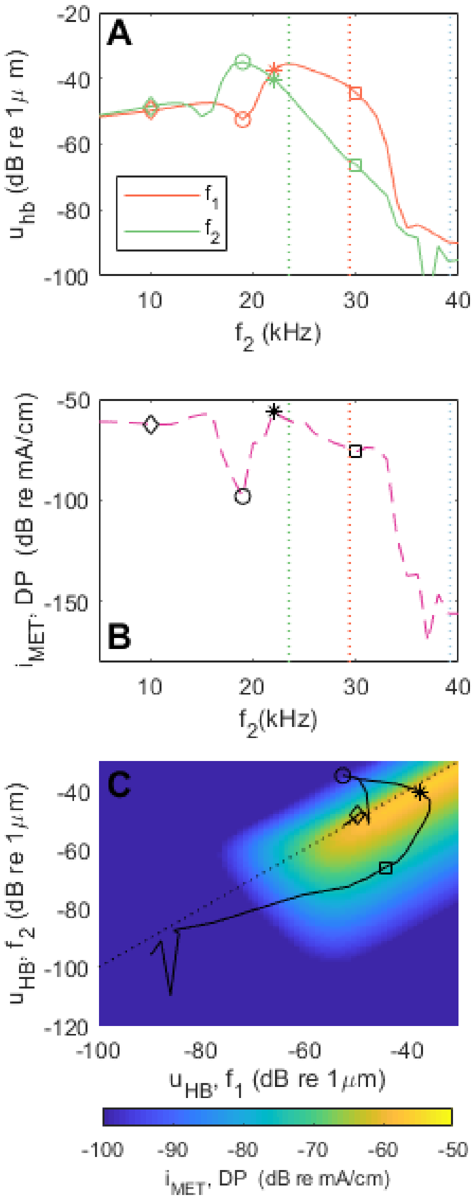

Figure S5. Origin of the notch in the eIDP response at 19 kHz in response to a two-tone stimulus with 80 dB SPL primaries. A: Level of the f_1 and f_2 components of the HB deflection predicted by the cochlear model at the 23.5 kHz tonotopic place in response to 80 dB SPL primaries. B: DP due to local nonlinear distortion predicted by Eq. (1) when the transduction channels are stimulated by vibrations of the HB at the f_1 and f_2 frequencies with the magnitude shown in panel A (this corresponds to the transduction current due to nonlinear distortion defined in section 4 below). C: Level of the DP in the transduction current as a function of the level of f_1 and f_2 components of the HB deflection. The black solid line corresponds to the levels predicted by the cochlear model in panel A for f_2 between 5 and 40 kHz . The diamond, circle, star, and square symbols correspond to the levels predicted at 10 kHz, 19 kHz, 22 kHz, and 30 kHz.

6. **Decomposition of voltage into the nonlinear distortion component and the component resulting from propagating fIDPs**

After application of the finite element methods, the governing equations for the electrical model shown in Fig. 1B can be written as the following nonlinear system of ODEs:

$$[c_e]\dot{\vec{V}} + [k_e]\vec{V} = -\vec{I}_{MET}(\vec{u}) - [C_{es}]\dot{\vec{u}}, \qquad (S6)$$

where the matrix $[C_{es}]$ arise due to Eq. 2, the vector $\vec{I}_{MET}$ is a vector of current sources due to mechano-electrical transduction, $[k_e]$ and $[c_e]$ are square matrices, $\vec{u}$ is the vector formed by assembling the structural degrees of freedom (i.e., displacements of BM and TM) at each longitudinal cross-section of the model and $\vec{V}$ is the vector formed by assembling the electrical degrees of freedom (electrical potentials) at each longitudinal cross-section of the model:

$$\vec{V} = [V_{sv}(x_1), V_{sm}(x_1), V_{ohc}(x_1), V_{st}(x_1), \dots, V_{sv}(x_N), V_{sm}(x_N), V_{ohc}(x_N), V_{st}(x_N)]^T, \qquad (S7)$$

where N is the number of longitudinal cross-sections of the model. The nonlinearity of the transduction channel is the source of nonlinear distortion in the model. As seen in Eq. (1), the transduction current is a nonlinear function of the HB deflection, $i_{hb}(x,t) = i_{hb}[u_{hb}(x,t)]$, where $u_{hb}(x,t)$ denotes the value of the HB deflection at location x and time t. Let $|U_{hb}(x,\omega)|$ and $\varphi_{hb}(x,\omega)$ be the amplitude and phase, respectively, of the FFT component of frequency ω of $u_{hb}(x,t)$. If harmonics of the primary components and other distortion products are neglected, $u_{hb}(x,t)$ is the sum of primary components and of the DP component of frequency $\omega_{DP} = 2\omega_1 - \omega_2$:

$$u_{hb}(x,t) \approx u_{hb}^{prim}(x,t) + u_{hb}^{(DP)}(x,t) \qquad (S8)$$

where $u_{hb}^{prim}(t)$ is the waveform of the HB deflection at the frequencies of the primary tones:

$$u_{hb}^{prim}(x,t) = |U_{hb}(x,\omega_1)| cos(\omega_1 t + \varphi_{hb}(x,\omega_1)) + |U_{hb}(x,\omega_2)|\, cos(\omega_2 t + \varphi_{hb}(x,\omega_2)) \qquad (S9)$$

and $u_{hb}^{(DP)}(x,t)$ is the waveform of the HB deflection at the DP frequency:

$$u_{hb}^{(DP)}(x,t) = |U_{hb}(x,\omega_{DP})|\, cos(\omega_{DP} t + \varphi_{hb}(x,\omega_{DP})) \qquad (S10)$$

The transduction current can be written as:

$$i_{hb}(x,t) = i_{hb}[u_{hb}(x,t)] = i_{hb}\left[u_{hb}^{prim}(x,t) + u_{hb}^{(DP)}(x,t)\right] \qquad (S11)$$

If the suppressive effects of the primaries on the DP voltage, and of the DP response on the primaries are neglected, the DP frequency component of the transduction current can be decomposed into the sum of two terms:

$$I_{hb}(x,\omega_{DP}) \approx I_{hb}\left[u_{hb}^{prim}(x,t), \omega_{DP}\right] + I_{hb}\left[u_{hb}^{(DP)}(x,t), \omega_{DP}\right] \qquad (S12)$$

The 1st term in this equation, $I_{hb}\left[u_{hb}^{prim}(x,t), \omega_{DP}\right]$, corresponds to the transduction current due to nonlinear distortion, while the 2nd term is the DP frequency component of the transduction current

induced by the vibrations of the HB at the DP frequency caused by the propagating fIDP waves, which we call the component resulting from propagating fIDPs in the manuscript. Because the current source due to somatic motility is a linear term ($[C_{es}]\vec{u}$), the somatic current only corresponds to a contribution induced by the vibrations of the OHC main body at the DP frequency. Eqs. (S6) and (S12) can be used to calculate the vector of electrical degrees freedom due to local nonlinear distortion,

$$\vec{V}_{distortion}(\omega_{DP}) = -(i\omega_{DP}[c_e] + [k_e])^{-1}\vec{I}_{hb}\left[u_{hb}^{prim}(t), \omega_{DP}\right] \qquad (S13)$$

Similarly, Eqs. (S6) and (S12) can be used to calculate the component resulting from propagating fIDPs:

$$\vec{V}_{DP\ ,propagating\ fIDPs}(\omega_{DP}) = -(i\omega_{DP}[c_e] + [k_e])^{-1}\left\{I_{hb}\left[u_{hb}^{(DP)}(t), \omega_{DP}\right] + i\omega_{dp}[C_{es}]\vec{U}(\omega_{DP})\right\} \qquad (S14)$$

The results of the decomposition of the total eIDP into the nonlinear distortion component and the component resulting from propagating fIDPs is shown in Fig. S6 for primary tones of level 40 to 80 dB SPL.

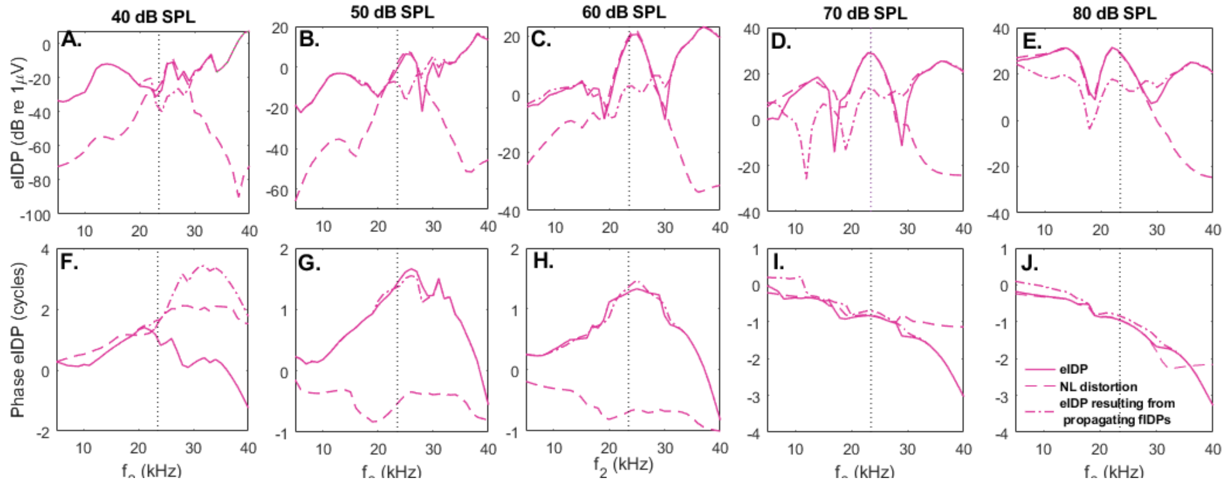

Figure S6. Amplitude and phase of the total eIDP, local nonlinear distortion eIDP and eIDP resulting from propagating fIDPs for primary tones of levels 40, 50, 60, 70 and 80 dB SPL.

7. Application of local damage to OHCs: To evaluate where the DPOAE originates from within the cochlea, local damage was applied by multiplying the conductance of the MET channel by $1 - d(x)$, where $d(x)$ is the following smooth function illustrated in Fig. S5 and given by:

$$d(x) = 0\ if\left|x - x_{damage}\right| \geq \frac{\Delta x}{2} + x_r \qquad (S15)$$

$$d(x) = \frac{1}{2}\left[1 - cos\left(\pi\frac{x - x_r + \frac{\Delta x}{2}}{x_r}\right)\right] if -\frac{\Delta x}{2} - x_r \leq x - x_{damage} \leq -\frac{\Delta x}{2} \qquad (S16)$$

$$d(x) = 1\ if\left|x - x_{damage}\right| \leq \frac{\Delta x}{2} \qquad (S17)$$

$$d(x) = \frac{1}{2}\left[1 - cos\left(\pi \frac{x - x_r - \frac{\Delta x}{2}}{x_r}\right)\right] if \frac{\Delta x}{2} \leq x - x_{damage} \leq \frac{\Delta x}{2} + x_r \qquad (S18)$$

where x_{damage} is the center of the damaged region. $d(x) = 0$ represents the baseline, healthy cochlea while $d(x) = 1$ represents a fully damaged location, where the MET current and nonlinearity are completely eliminated. Applying local damage in the model causes total elimination of the MET current over a well-defined, and potentially narrow, spatial region. Any abrupt change in the spatially dependent cochlear properties will cause reflections; to minimize these reflections, the function $d(x)$ has a smooth transition between the "undamaged" baseline regions to the "damaged" regions. However, despite this smooth transition, some reflection is still observed when damage is applied at or slightly apical to the DP tonotopic place (see section 8 of the Supplementary Information).

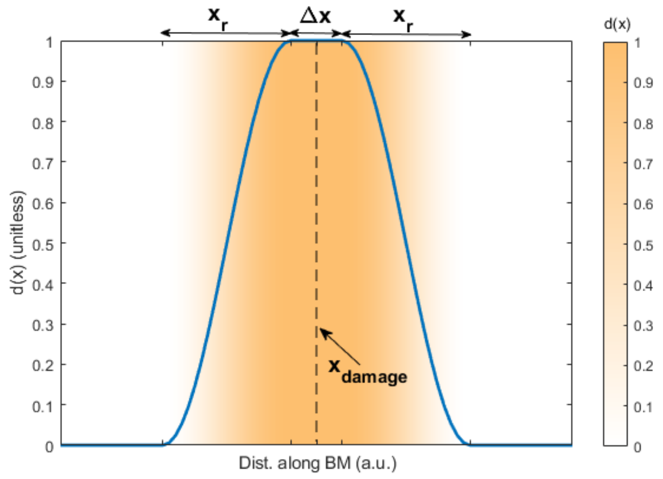

Figure S7. Sketch of the damage function, d(x). Numerical results shown in the manuscript were obtained using $\Delta x = 200$ μm *(which corresponds to 20 sections of OHCs) and* $x_r = 500$ μm.

8. Quantification of the reflection introduced by applying the local OHC damage

When the local damage is applied apical to the DP best place, an enhancement in DPOAE is observed in response to low level primaries (see Fig. 5G at around 0.48 cm). Analysis described in this section demonstrates that this local enhancement does not imply that the baseline model predicts that some of the DPOAE originates from locations apical or close to the DP best place.

Even though a smoothly varying damage function is used, introducing damage causes a relatively abrupt change in model activity, such that the damage creates a reflection mechanism for forward propagating IDP waves. To evaluate the effects of such a reflection mechanism in the model, a low-level pure tone with the same frequency (12 kHz) as the DP in the two-tone simulation of Fig. 5 was delivered to a baseline model and a model with local damage. The center of the damage location was varied from the base to apical to the 12 kHz best place. The reflection of forward traveling wave can be characterized by the apical reflectance, R_a, which is defined as the ratio of outward (reverse) traveling wave, P_{out}, to inward (forward) traveling waves $P_{in}(f)$ at the stapes:

$$R_a(f) = \left.\frac{P_{out}(f, P_{in})}{P_{in}(f)}\right|_{stapes} \qquad (S\ 19)$$

Once waves are reflected inside the cochlea, they propagate in the reverse direction towards the stapes where they can be again reflected due the impedance mismatch between the cochlea and middle ear, such that multiple internal reflections are present in the cochlea. By summing up the multiple reflection component within the cochlea, we can express the total reflection due to damage as a function of the apical reflectance:

$$P_{reflection} = P_0 G_{mert} \frac{R_a(1+R_{st})}{1-R_a R_{st}} \tag{S20}$$

Where P_0 is the ear canal pressure at the stimulus frequency, G_{mert} is the round trip middle ear pressure transfer function and R_{st} being the reflection coefficient for retrograde waves at the stapes (commonly called the stapes reflectance) [6]. $P_{reflection}$ is the reflected pressure component, defined here as the pressure difference between the baseline model and model with local damage. Eq. S19 can be used to calculate R_a.

As shown in Fig. S8, the apical reflectance is maximum when damage is applied at the same location as where the DPOAE is predicted to see the maximum enhancement in Fig. 5. According to cochlear literature, reflection is expected to be maximum around the peak location [7]. It can be therefore counter-intuitive to observe that the maximum apical reflectance occurs when the center of the damage region is significantly apical to the peak location. However, the onset of the damage is $x_r + \Delta x/2 = 60\ \mu m$ basal to the center of the damage region. As shown in Fig. S8B, for the damage that results in the maximum apical reflectance, the onset of the damaged region is pretty much at the peak location. While this damage has limited influence on the peak ST pressure (because cochlear amplification occurs basal to the peak), it introduces a reflection mechanism.

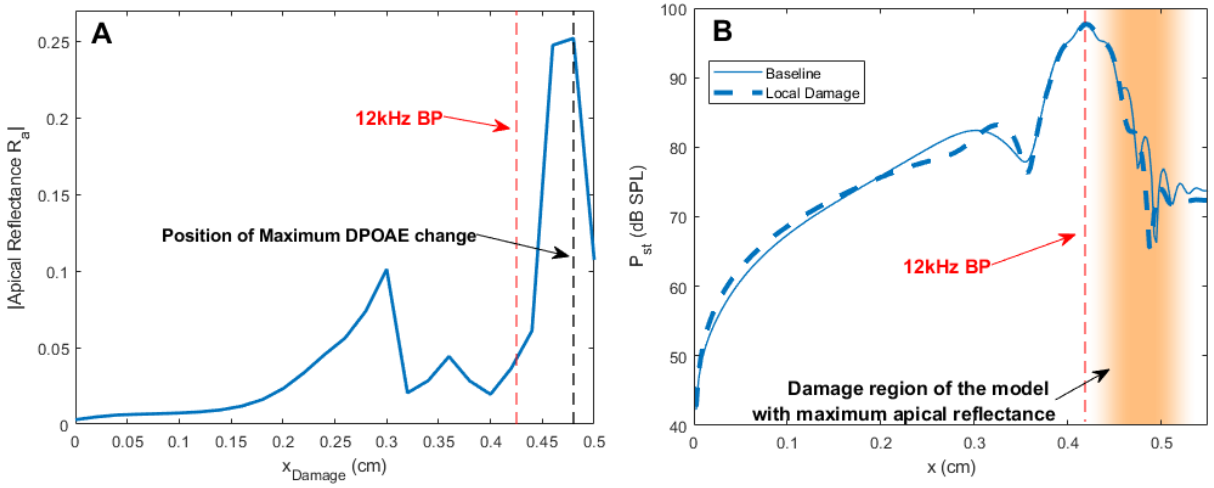

Fig.S8. A. Magnitude of the apical reflectance, $|R_a|$, as a function of center of the damaged region in response to a pure tone of frequency 12 kHz. The 12 kHz tonotopic place is shown in a red dashed line; the black dashed line corresponds to the location of maximum DPOAE enhancement in Fig 4. B. ST pressure predicted by model in response to low level pure tone. The damage region of the model with maximum apical reflectance is shown in the shaded orange area. The thick dashed line corresponds to the model with the damage applied as shown in shaded orange area.

9. Model parameters

Table S1. Mechanical parameters of the cochlear model. (x is the longitudinal distance from the stapes expressed in cm)

Param.	Description	Value
K_{bm}	BM stiffness	$18.4\exp(-7.54x) \times 10^5\ N/m^2$
D_{xx}	BM plate bending stiffness (xx)	$10^{-10}\exp(-0.5x)\ N.m$
D_{xy}	BM plate bending stiffness (xy)	$10^{-10}\exp(-0.5x)\ N.m$
D_{shear}	BM plate bending stiffness (shear)	$4.3\exp(-0.5x) \times 10^{-11} N.m$
K_{tms}	TM shear stiffness	$2.77\exp(-1.32x^2 - 6.43x) \times 10^5 N/m^2$
K_{tmb}	TM bending stiffness	$3.84\exp(-7.54x) \times 10^4 N/m^2$
K_{rl}	RL stiffness	$2.78\exp(-7.54x) \times 10^3 N/m^2$
K_{ohc}	OHC stiffness	$5.07\exp(-7.54x) \times 10^3 N/m^2$
K_{hb}	HB stiffness	$291\exp(-7.54x) \times 10^3 mN/m^2$
M_{bm}	BM mass	$2.8 \times 10^{-7} kg/m$
M_{tms}	TM shear mass	$3.58\exp(1.58x) \times 10^{-6} kg/m$
M_{tmb}	TM bending mass	$1.46\exp(2.15x) \times 10^{-6} kg/m$
c_{bm}	BM damping coefficient	$8.5 * 10^{-2} Ns/m^2$
c_{hb}	HM damping coefficient	$\eta_f \frac{L_{tm}}{3L_{hb}}$, where $\eta_f = 1.0 \times 10^{-3} N.s/m^2$ is the viscosity of the fluid
c_{tmb}	TM bending damping coefficient	$0.1\ N.s/m^2$
c_{tms}	TM shear damping coefficient	$3 \times 10^{-2} Ns/m^2$
G_{tm}	TM modulus	$7.0\exp(-3.75x)\ kPa$
η_{tm}	TM viscosity	$0.03\ \mathrm{Pa.s}$
ρ_f	Fluid density	$1000\ \mathrm{kg}/m^3$

Table S2. Electrical parameters of the model (*x* is expressed in cm). The model assumes 1000 rows of OHCs per cm.

Param.	Description	Value in Model	Reference	Value in Reference
G_a^{max}	Saturating HB conductance	Interpolated from<br>86.5 nS at *x* = 0 cm<br>83.6 nS at *x* = 0.13 cm<br>66.5 nS at *x* = 0.224 cm<br>49.8 nS at *x* = 0.32 cm<br>34.1 nS at *x* = 0.44 cm<br>17.1 nS at *x* = 0.67 cm<br>3.82 nS at *x* = 1.12 cm	Based on Johnson *et al.*, 2011	~ 36 nS for CF=10 kHz (*x*=0.47 cm)<br>~ 12 nS for CF=0.4 kHz (*x*=1.12 cm)
R_{mg}	SM-ground resistance	27 Ω.m	D. Strelioff 1973 (R6)	~27 Ω.m
R_{tl}	ST-ground resistance	0.25 Ω.m	Assumed	N/A
λ_{st}	ST space constant	134 μm	Fridberger et al., 2004<br>Dong and Olson, 2013	42 μm<br><br>80 μm
R_{vm}	Resistance from SV to SM	25 Ω.m	D. Strelioff 1973 (R2)	~27 Ω.m
r_{sm}	SM longitudinal resistance	2,000 MΩ/m	Teal et al., 2016	~2000 MΩ/m
R_{vl}	SV-ground resistance	10 Ω.m	D. Strelioff 1973 (R1)	~10 Ω.m
r_{sv}	SV longitudinal resistance	3 MΩ/m	D. Strelioff 1973 (R8)	~3 MΩ/m
C_m	basolateral capacitance (per OHC)	17.45*x* pF	Johnson *et al.*, 2011	~5 pF for CF=10 kHz<br>~ 18 pF for CF=0.4 kHz
G_m	OHC basolateral conductance (per OHC)	192-148.9*x* nS	Johnson *et al.*, 2011 (based on cutoff time constant)	~188 nS for CF=10 kHz<br>~33.9 nS for CF=0.4 kHz
ε_3	OHC electromechanical coupling coefficient (per unit length)	(-10.4+3.6*x*) µN/m/mV	Iwasa 1997	-10 µN/m/mV
R_a^0	OHC apical resistance	58.1 exp(2.05*x*) Ω.m	assumed	
C_a	apical capacitance (per OHC)	0.5 pF	Dallos and Evans, 1995	0.52 pF at *x*=0.5cm (based on $C_a/C_m = 0.06$)
ΔV_{hb}^0	resting value of potential difference between SM and intracellular OHC potential	150-10*x* mV	Meaud 2012	150-10*x* mV
P_0^s	resting probability	0.4	assumed	
f_{gs}	single channel gating force in the tip link direction	40 pN	assumed	

Middle ear parameters: the following values are chosen for the mass, damping and stiffness coefficients of the middle ear, respectively: M_{me} = 3.148×10^{-9} kg, C_{me} = 0.9576 Ns/m, K_{me} = 7,750 N/m. When coupled to the cochlear model, the middle ear reverse middle ear impedance is predicted to have the magnitude and phase shown in Fig. S9.

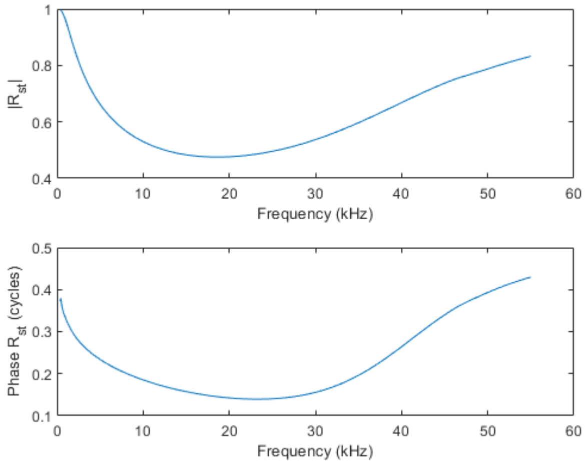

Figure S9. Amplitude and phase of the stapes reflectance when the cochlear model is coupled to the 1 degree-of-freedom model.

<u>References</u>

1. Dong, W. and E.S. Olson, *Detection of cochlear amplification and its activation.* Biophys J, 2013. **105**(4): p. 1067-78.
2. Johnson, S.L., et al., *Prestin-driven cochlear amplification is not limited by the outer hair cell membrane time constant.* 2011. **70**(6): p. 1143-1154.
3. Ren, T., W. He, and D. Kemp, *Reticular lamina and basilar membrane vibrations in living mouse cochleae.* Proc Natl Acad Sci U S A, 2016. **113**(35): p. 9910-5.
4. Fallah, E., C.E. Strimbu, and E.S. Olson, *Nonlinearity and amplification in cochlear responses to single and multi-tone stimuli.* Hear Res, 2019. **377**: p. 271-281.
5. Lee, H.Y., et al., *Two-Dimensional Cochlear Micromechanics Measured In Vivo Demonstrate Radial Tuning within the Mouse Organ of Corti.* Journal of Neuroscience, 2016. **36**(31): p. 8160-8173.
6. Shera, C.A., *Mammalian spontaneous otoacoustic emissions are amplitude-stabilized cochlear standing waves.* J Acoust Soc Am, 2003. **114**(1): p. 244-62.
7. Shera, C.A. and J.J. Guinan, Jr., *Stimulus-frequency-emission group delay: a test of coherent reflection filtering and a window on cochlear tuning.* J Acoust Soc Am, 2003. **113**(5): p. 2762-72.
